# High-Load Reovirus Infections Do Not Imply Physiological Impairment in Salmon

**DOI:** 10.3389/fphys.2019.00114

**Published:** 2019-03-13

**Authors:** Yangfan Zhang, Mark P. Polinski, Phillip R. Morrison, Colin J. Brauner, Anthony P. Farrell, Kyle A. Garver

**Affiliations:** ^1^Faculty of Land and Food Systems, The University of British Columbia, Vancouver, BC, Canada; ^2^Aquatic Diagnostics and Genomics Division, Pacific Biological Station, Fisheries and Oceans Canada, Nanaimo, BC, Canada; ^3^Department of Zoology, The University of British Columbia, Vancouver, BC, Canada

**Keywords:** piscine orthoreovirus, salmon, cardiorespiratory performance, heart inflammation, viremia, nucleated erythrocytes

## Abstract

The recent ubiquitous detection of PRV among salmonids has sparked international concern about the cardiorespiratory performance of infected wild and farmed salmon. Piscine orthoreovirus (PRV) has been shown to create substantial viremia in salmon by targeting erythrocytes for principle replication. In some instances, infections develop into heart and skeletal muscle inflammation (HSMI) or other pathological conditions affecting the respiratory system. Critical to assessing the seriousness of PRV infections are controlled infection studies that measure physiological impairment to critical life support systems. Respiratory performance is such a system and here multiple indices were measured to test the hypothesis that a low-virulence strain of PRV from Pacific Canada compromises the cardiorespiratory capabilities of Atlantic salmon. Contrary to this hypothesis, the oxygen affinity and carrying capacity of erythrocytes were unaffected by PRV despite the presence of severe viremia, minor heart pathology and transient cellular activation of antiviral response pathways. Similarly, PRV-infected fish had neither sustained nor appreciable differences in respiratory capabilities compared with control fish. The lack of functional harm to salmon infected with PRV in this instance highlights that, in an era of unprecedented virus discovery, detection of viral infection does not necessarily imply bodily harm and that viral load is not always a suitable predictor of disease within a host organism.

## Introduction

Animal viruses are expected to inflict harm to the host cells they infect, either as a direct result of infection or as a product of host-directed apoptosis (Kaminskyy and Zhivotovsky, 2010). As a consequence, all animal viruses can be defined as putatively pathogenic, varying only in their potential virulence (i.e., their relative ability to cause damage) (Pirofski and Casadevall, 2012). In cases where highly virulent viruses are linked to serious disease, the presentation of severe clinical symptoms such as morbidity or death clearly represent states of reduced fitness and are usually conditional on the quantity of virus produced during infection (Griffiths, 1999). However, harm inflicted to animals experiencing infections of either low intensity or low virulence becomes far less clear, given the physiological plasticity animals can display and the potential for pathogenic viruses to provide host benefits; for example, protection against a more serious transmissible or non-transmissible disease (Tan et al., 2012; Cadwell, 2015; Villalona-Calero et al., 2016).

Piscine orthoreovirus (PRV) is the most recently accepted member of the Orthoreovirus genus. It has a pervasive and global distribution in salmon, including both wild and farmed populations, and has been detected in nearly all salmonid species (Garseth et al., 2013; Marty et al., 2015; Olsen et al., 2015; Takano et al., 2016; Morton et al., 2017; Julio et al., 2018). Phylogenetic comparisons currently suggest there are three subtypes of PRV (PRV1, PRV2, and PRV3) which loosely correspond to geographical and/or host species divisions (Kibenge et al., 2013; Olsen et al., 2015; Takano et al., 2016). Nevertheless, all three subtypes have a proclivity for infecting erythrocytes and can generate extensive systemic infections.

The virulence of PRV, like most reoviruses, appears to be generally low and highly dependent on virus, host, and/or environmental factors (Garver et al., 2016; Polinski et al., 2019). Nevertheless, PRV1 has been demonstrated as an etiological component of a disease known as heart and skeletal muscle inflammation (HSMI) in farmed Atlantic salmon of Norway (Wessel et al., 2017), which is currently one of the transmissible diseases most affecting Atlantic salmon production in that country ^1^ – the largest Atlantic salmon producer in the world. In Pacific Canada, PRV1 has also been suggested to be involved in a jaundice/anemia syndrome of farmed Chinook salmon (Di Cicco et al., 2018), although its role in this relatively rare disease is far from clear (Garver et al., 2015). In Japan, PRV2 has been associated with an anemic condition of farmed Coho salmon, known as erythrocytic inclusion body syndrome (EIBS) (Takano et al., 2016), while in Europe, PRV3 is associated with an HSMI-like condition in farmed rainbow trout (Olsen et al., 2015). High morbidity has been attributed to both EIBS and HSMI in some farmed populations.

Given that erythrocytes are primary targets for PRV infection and the heart is a principal organ associated with PRV disease (e.g., HSMI), it has been suggested that all PRV infections lead to respiratory impairments (Olsen et al., 2015; Takano et al., 2016; Lund et al., 2017; Di Cicco et al., 2018) and are of sufficient severity that if wild salmon become infected they might not be able to swim back to their spawning areas because of a presumed reduction in respiratory capabilities (Garseth et al., 2013; Morton et al., 2017; Di Cicco et al., 2018). This has sparked international concern for PRV’s potential to cause cardiorespiratory harm to both wild and farmed salmon populations. Even in the absence of direct tests of such respiratory harm, a Canadian Federal Justice has already ruled that PRV ‘may be harmful to the protection and conservation of fish’ [Morton v. Canada (Fisheries and Oceans) 2015 FC 575].

However, experimental challenge with a PRV1 subtype from Pacific Canada has failed to produce noteworthy pathology, anemia, or other signs of disease despite causing extreme viremia (>10^9^ PRV genome copies per mL host blood) (Garver et al., 2016; Polinski et al., 2019). While such high-load systemic infections exhibiting no-to-low virulence are rare in virus-animal relationships (if not unprecedented), these findings present an alternative possibility that at least some subtypes of PRV may have found a near commensal opportunity in targeting erythrocytes; which although remain nucleated in salmon, have minimal transcriptional requirements following maturation and have unknown capacities for communicating with immune competent cells (Lund et al., 2000; Phillips et al., 2000). Nevertheless, it is also unknown as to whether there is physiological harm associated with the minor amounts of cardiac inflammation, transient activation of virus recognition pathways, and erythrocytic inclusion bodies observed during these low-virulent PRV infections (Polinski et al., 2019). This remains a substantial knowledge gap in the foundational understanding of the true virulence potential of PRV.

Here we tested the hypothesis that the low-virulence subtype of PRV1 endemic to Pacific Canada compromises the respiratory capabilities of Atlantic salmon. To accomplish this, we took advantage of the high-load PRV laboratory challenge model developed in Pacific Canada (Garver et al., 2016; Polinski et al., 2019) and combined it with a recently developed and highly comprehensive integrated respiratory assessment paradigm (IRAP) to measure a suite of indices related to the respiratory performance of fish (Zhang et al., 2016, 2017, 2018). We further measured the oxygen affinity properties of erythrocytes during infection. Using IRAP in concert with molecular and histopathological tools for assessing viral kinetics and host-pathogen interactions, our comprehensive examination targeted all phases of the time-course of the PRV infection (early entry and dissemination, peak viremia, and latent persistence). We also incorporated an extreme acute hypoxic challenge into the late persistence phase of infection so as to maximize our likelihood for identifying PRV-associated respiratory harm.

## Materials and Methods

This study was conducted on a population of Pacific-domesticated Mowi-McConnell strain Atlantic salmon (Withler et al., 2005) developed specifically for use by the British Columbia Atlantic salmon farming industry. A cohort was obtained from a single commercial hatchery on Vancouver Island, Canada, and transported to the Fisheries and Oceans Canada Pacific Biological Station (49.2106° N, 123.9554° W) where all experimental trials were conducted. Experiments were carried out under the permission and guidelines of the Canadian Council on Animal Care (CCAC) Pacific Region Animal Care Committee (no: 16–019). A pre-transport screening of 20 fish did not detect PRV by quantitative PCR (qPCR), nor was any other virus propagated on CHSE-214 or EPC diagnostic fish cell lines. Fish were reared for 4 months in 8°C municipal dechlorinated freshwater before being transitioned to sand-filtered, UV-irradiated, 11°C seawater (28–32 ppt) over a 4 week period. Two hundred and forty fish (∼75 g body mass by bulk weight estimate) were divided equally into six 350 L circular experimental tanks (40 fish per tank) supplied with 15 L per min flow-through, sand-filtered, UV-irradiated 11°C seawater and held for 10–20 days prior to challenge and first IRAP assessments (Zhang et al., 2019). Experiments began after seawater transfer because of the belief that this is when Atlantic salmon most often develop HSMI in a field setting (Lovoll et al., 2012). Similarly, experiments were conducted between 10–12°C in this study because PRV associated disease has manifested at these temperatures in both laboratory and field environments (Kongtorp et al., 2006; Wessel et al., 2017). Fish were fed EWOS dry pellet rations at 0.5% body weight per day throughout the study and dissolved oxygen was maintained above 90% air saturation (>8.2 mg per L).

### Experimental Infection

An isolation of PRV from a commercial freshwater salmon hatchery on Vancouver Island, Canada (designated 16-005ND) has provided a consistent well-characterized laboratory model for inducing high-load viremia in the Mowi-McConnell strain of Atlantic salmon following intraperitoneal (ip) injection (Garver et al., 2016; Polinski et al., 2019). This isolate is also representative of the PRV1 subtype which is widely and naturally found in both wild and farmed salmon of western North America (Kibenge et al., 2013; Siah et al., 2015). To ensure physiological harm caused by PRV could be properly differentiated, two treatment groups were tested periodically during a 21-week time course: (i) a blood control (BC) treatment in which sonified and clarified Atlantic salmon blood diluted in Hank’s balanced salt solution (HBSS) was injected (total injection volume 0.1 mL) and (ii) a PRV 16-005ND (PRV) treatment in which sonified and clarified PRV-infected Atlantic salmon blood diluted with HBSS at a final concentration of 3.25 × 10^7^ copies PRV L1 reverse-transcribed genome segments was injected (total injection volume 0.1 mL). In addition, a saline control (SC) treatment group was tested following a similar injection of HBSS to (i) provide basic control for time-dependent changes in respiratory physiology over the 21-week experiment and (ii) identify any effects associated with the injection of a foreign blood homogenate.

Inoculates were prepared and administered as previously described (Polinski et al., 2019). Briefly, blood of commercially produced salmon that had been frozen at −80°C was thawed, diluted 1:10 in HBSS, sonified for 80s in 10s bursts with 30s rests on ice and clarified via centrifugation at 2000 × *g* for 5 min at 4°C. Inoculate sourced from PRV infected fish was then passed four times through 30–50 *g* Atlantic salmon (3–4 week incubation period per pass) to increase the inoculating dose since PRV cannot currently be propagated *in vitro.* Inoculates were identically prepared between each passage. Each treatment was administered to fish that were evenly distributed into two replicate experimental tanks. Starting with SC, administration of treatments was conducted in 5-day increments so IRAP assessments, which in our facility could only be conducted on one treatment group at any given time, would be time-matched relative to days post-challenge (dpc) rather than calendar day (Zhang et al., 2019). Because IRAP assessments lasted 5 days, data are reported as the week post-challenge (wpc) while data from tissues collected on specific days are reported as day post-challenge (dpc).

### Measuring Respiratory Performance

All oxygen removed from water by a fish must be transported by the blood to tissues with the exception of minor quantities used directly by skin and gill (Farrell et al., 2014). By far, the majority of this internally transported oxygen is bound to hemoglobin in erythrocytes, which are pumped by the heart. Therefore, the least invasive method of detecting cardiorespiratory impairments in fish is through respirometry, which was the assessment approach used here. Fish were also sacrificed after testing to sample blood and directly measure the oxygen affinity properties of the erythrocytes.

The experimental protocol used for IRAP measured 14 respiratory indices and provides a near comprehensive assessment of fish respiratory capabilities. Four indices directly assess aerobic capabilities: standard metabolic rate (SMR) and maximum oxygen uptake (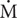
O_2max_), as well as the derived indices of absolute aerobic scope (AAS = 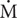
O_2max_ – SMR) and factorial aerobic scope (FAS = SMR/ 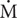
O_2max_). Two indices directly assess the capability of fish to recover from exhaustion: excess post-exercise oxygen consumption (EPOC) and length of time to recover (EPOC_dur_). Three indices assess levels of fish activity and agitation: routine metabolic rate (RMR), time spent above 50% maximum oxygen consumption rate (T_0.5
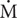
O2max_) and time spent above 80% maximum oxygen consumption rate (T_0.8
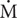
O2max_). Lastly, five indices assess hypoxia tolerance and anaerobic capabilities: critical oxygen level (O_2crit_), incipient lethal oxygen saturation (ILOS), scope for oxygen deficit (SOD), factorial scope for oxygen deficit (FSOD), and accumulated oxygen deficit (AOD) (see Supplementary Material for detailed calculations).

Integrated respiratory assessment paradigm assessment was performed with 8 fish simultaneously using an 8-chamber intermittent-flow respirometry system (Zhang et al., 2016, 2017, 2018). The water in the respirometry system was maintained at 11°C (± 0.5) by being immersed in a 600 L flow-through water bath of 11°C seawater. An IRAP assessment was conducted on 8 fish per treatment group (4 from each replicate holding tank) during the 1st week (early viral replication and dissention), the 4th week (peak viremia), the 10th week (early viral latency period with minor heart inflammation), and the 18th week (late viral latency period with minor heart inflammation) post-challenge. An additional IRAP test was conducted during the 21st wpc using the same fish that had been assessed at week 18. All IRAP tests usually ended with fish experiencing acute hypoxia and loss of equilibrium where after they were humanely sacrificed for tissue recovery; but all fish were revived at 18 wpc and held for a further 3 weeks following this severe acute hypoxic stress with the expectation that the hypoxic stress would exacerbate the cardiorespiratory impairments induced by PRV.

In all IRAP assessments, fish were first fasted for 72 h and then individually hand chased for 7 min in a 20 L bucket. At exhaustion, fish were exposed to a standardized 1 min air exposure before being placed into each respirometer to measure 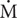
O_2max_ and subsequent recovery. During the 1st hour, as peak oxygen uptake was subsiding, water cycling conditions through each respirometry chamber consisted of a 25 s flush with fully aerated 11°C seawater, 70 s sealed stabilization, and 110 s sealed oxygen uptake monitoring period. After 1 h, cycling conditions were modified to a 50 s flush, 115 s sealed stabilization, and 345 s oxygen uptake monitoring period which maintained > 85% air saturation within the respirometry chambers at all times. Fish were kept in this normoxic state without disturbance for 4 days during which time oxygen uptake was recorded approximately every 10 min to ensure that SMR was reliably measured (Chabot et al., 2016). Afterward, the respirometry system was made hypoxic (40% air saturation) over a 45-min period using nitrogen gas supplementation. Oxygen was further reduced at a rate of 0.15% per min until each fish lost its dorso-ventral equilibrium (LOE) and the percent air saturation at LOE was noted. At 1, 4, 10 and 21 wpc fish were immediately removed from the respirometer and killed by blunt head trauma. At 18 wpc, fish were recovered in a bath of fully aerated seawater and replaced into their experimental holding tanks. Marked recapture of these fish was facilitated by unique passive integrated transponder (PIT) tags that had previously been implanted at approximately 15 wpc into their intra-peritoneal cavities (Zhang et al., 2019).

### PRV-Associated Sample Collection and Processing

Blood and tissue samples were collected from all fish assessed with IRAP as well as from an equal number (*n* = 8) of non-assessed fish directly from the holding tanks at 4, 25, 67, and 148 dpc that corresponded to the termination of an IRAP (1, 4, 10 and 21 wpc). Additional fish (*n* = 3 per treatment group) were sampled at 14, 39 and 53 dpc to monitor PRV loads. Blood (1 mL) was collected via caudle puncture and a 100 μL aliquot immediately frozen in liquid nitrogen for PRV screening and host gene expression analysis by qPCR as previously described (Polinski et al., 2019). Hematocrit was determined on a 10 μL aliquot transferred to a sodium-heparin treated Fisherbrand^TM^ micro-hematocrit tube and spun at 15,000 × *g* for 10 min. A 5–10 μL aliquot was smeared on a glass microscope slide for cytoplasmic inclusion body visualization (Finstad et al., 2014). Remaining blood was transferred to a heparinized vacutainer held on ice (∼24 h) from which oxygen equilibria curves were determined using a custom microplate-based, parallel assay, multi-cuvette tonometry cell system maintained at 11.5°C as described by Lilly et al. (2013). Oxygen binding was determined at a constant carbon dioxide fraction of 0.25% (1.90 mmHg) over 9 stepwise increments of increasing oxygen balanced with nitrogen. Blood pH was measured following 1 h incubation in a rotating glass tonometer held at 11.5°C with 21% oxygen and 0.25% carbon dioxide balanced with nitrogen using two microelectrodes (16–705 and 16–702; Microelectrodes Inc., Bedford, NH, United States). Hemoglobin concentration (Hb) was measured by the cyan-methemoglobin method using Drabkin’s Reagent (Sigma-Aldrich, St. Louis, MO, United States) and a heme-based extinction coefficient of 11.01 mmol^−1^ cm^−1^ at a wavelength of 540 nm (Volkel and Berenbrink, 2000).

Approximately 200 mg of skeletal muscle (red and white) was excised near the left lateral line of each fish at mid-body and preserved in 10% neutral buffered formalin for histopathology. Hearts were bisected longitudinally and one half preserved in 10% neutral buffered formalin for histopathology while the other half was immediately frozen in liquid nitrogen for PRV screening and host gene expression (qPCR) analyses (Polinski et al., 2019). Normalized quantities were scaled to the minimum value for each gene prior to analysis and a five-step fourfold dilution series of pooled cDNA was run on all plates to estimate amplification efficiency and provide inter-run calibration where necessary. Histopathological examination was conducted blind to the treatments by two board certified pathologists at the BC Ministry of Agriculture Animal Health Center (Abbotsford, BC, Canada), as previously described (Marty et al., 2015; Garver et al., 2016; Polinski et al., 2019). A detailed scoring report of this histopathology evaluation is found in Zhang et al. (2019).

### Statistical Analysis

Median pathology scores were assessed across time, as well as at individual time points between BC and PRV treatment groups and between fish exposed to IRAP assessments versus those taken directly from the experimental holding tank, by Mann–Whitney *U*-tests without *p*-value multiplicity adjustment. Transcriptional expression of host immune genes were compared between BC and PRV treatment groups at each time point using two-way ANOVA and Tukey’s HSD multiple comparison tests of log transformed data normalized to β-actin – a gene previously demonstrated to have stable expression in blood and kidney following PRV infection of salmon (Garver et al., 2016; Polinski et al., 2016). Treatment effect (BC vs. PRV) on respiratory performance indices and erythrocyte oxygen transport indices were compared at each discrete sampling event up to 18 wpc (128 dpc) by two-way ANOVA and four multiple comparison tests: Fisher’s LSD (no family-wise *p*-value multiplicity adjustment), Bonferroni (family-wise confidence 0.05), Sidak (family-wise confidence 0.05), and Holm-Sidak (family-wise confidence 0.05). Time-dependent changes in baseline respiratory performance indices of the SC treatment group were assessed with one-way ANOVA followed by Tukey’s HSD multiple comparison tests. The effect of blood homogenate injection (SC vs. BC) was assessed by two-way ANOVA and Fisher’s LSD, Bonferroni, Sidak, and Holm-Sidak multiple comparison tests. Because some but not all control (BC) fish developed mild heart pathology, the effect of heart pathology on respiratory performance of BC fish was assessed relative to fish without heart pathology (independent of time) by a Students *T*-test for each index with a family-wise confidence of 0.05 applied using the Holm-Sidak method. A repeated measure two-way ANOVA was used to compare all data collected at 21 wpc (IRAP) and 148 dpc (erythrocyte oxygen transport) along with Fisher’s LSD, Bonferroni, Sidak, and Holm-Sidak multiple comparison tests in association with primary assessment data collected at 18 wpc (IRAP) and 128 dpc (erythrocyte oxygen transport) indices. Given that T_0.5
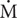
O2max_, T_0.8
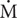
O2max_, hemoglobin saturation at 21% oxygen (Hb_21%O2_) and hematocrit data were proportionally constrained between 0 and 1, an arcsine transformation was applied to these data prior to comparisons. pH was compared in its untransformed variable (hydrogen ion concentration) as previously suggested (Murphy, 1982). All analyses were conducted using GraphPad Prism 6 or R v. 3.3.3 software.

## Results

### PRV Viremia and Its Associated Pathology Following Inoculation

**FIGURE 1:**
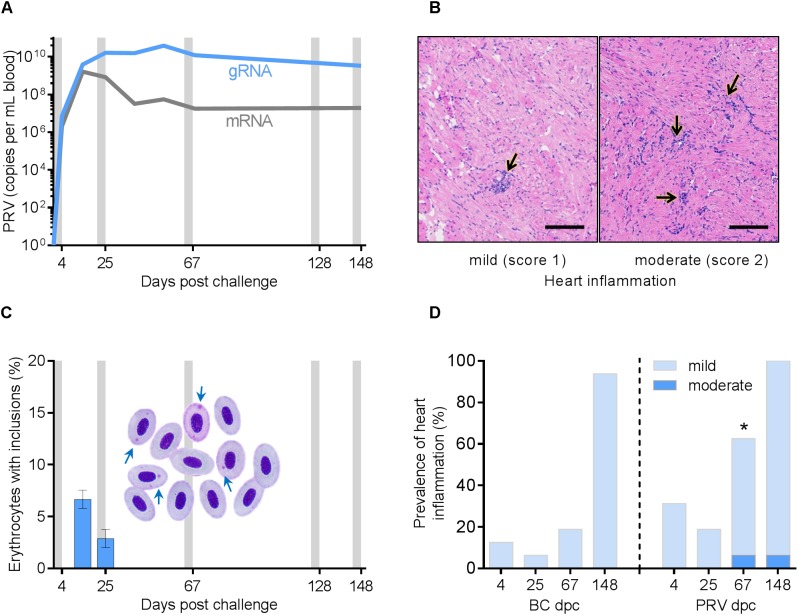
Piscine orthoreovirus viremia and associated pathology in experimentally infected Mowi-McConnell Atlantic salmon. **(A)** Systemic blood load of PRV L1 double-stranded genomic RNA (gRNA) segments and single-stranded messenger RNA (mRNA) as determined by qPCR following reverse transcription (mean ± SEM.; *n* = 3 or 16 per time point). Gray bars indicate periods of respirometry assessment. **(B)** Lymphohistiocytic endocarditis (arrows) of mild intensity (left panel) common to both BC and PRV challenged groups and of moderate intensity (right panel) present in only two PRV infected individuals. Scale bar = 100 μm. **(C)** The number of erythrocytes with cytoplasmic viral inclusion bodies (arrows) per 100 cells (mean ± s.e.m.; *n* = 8 per time point). **(D)** Prevalence of heart inflammation in BC and PRV injected groups (*n* = 16 per time point). ^∗^*p* < 0.05 increase of median inflammation severity score for PRV challenged fish relative to time matched BC controls.

Intraperitoneal injection of PRV generated an anticipated high-load viremia in Atlantic salmon as evidenced by PRV transcriptional blood loads reaching a peak by 25 dpc at approximately 2.0 × 10^10^ mean reverse transcribed gRNA copies per mL of host blood and maintained above 3.0 × 10^9^ mean copies per mL for the remainder of the study (Figure 1A). Evidence of viral protein production was identified to be proportionally large (mean 15 ± 2% s.e.m. single-stranded PRV mRNA relative to double-stranded genomic PRV RNA) during the early to peak infection period (4–25 dpc), but became substantially reduced (to approximately 0.5 ± 0.1% single stranded mRNA) after peak viral loads were reached (Figure 1A). Cytoplasmic inclusions characteristic of reovirus factories occurred transiently in up to 8% of erythrocytes within PRV infected salmon (Figure 1C). While mild heart and/or skeletal muscle inflammation was diagnosed in PRV-infected salmon, a comparable prevalence and severity of heart inflammation occurred in the BC-treatment group (*p* > 0.39) at 4, 25 and 148 dpc (Figure 1D and Supplementary Figure 1). SC fish were not considered for histology in this study and therefore it is unknown as to whether or not injection of blood homogenate material alone was responsible for generating at least some mild heart inflammation in BC fish. Nevertheless, PRV-infected fish had a higher cumulative median severity score for heart inflammation (*p* = 0.02) at 67 dpc (Figure 1D) and the only fish with moderate heart inflammation (2 fish) or skeletal muscle inflammation (1 fish) were in the PRV treatment group (Figure 1B and Supplementary Figures 2, 3). Similarly, while low prevalence of mild skeletal muscle inflammation, scale pocket inflammation, and myocardial degeneration of skeletal muscle also occurred, these histological changes were not specific to PRV-infected salmon (*p* > 0.27). The significantly (*p* = 0.01) increased accumulation of skeletal muscle degeneration after IRAP testing (7 of 64 IRAP fish) compared with fish from the experimental holding tanks (0 of 64 IRAP fish) was independent of PRV treatment. A similar independence from PRV was the significant reduction (*p* = 0.003) in heart inflammation for salmon that underwent an IRAP assessment (Supplementary Figure 1).

### Host Transcriptional Responses to Viral Infection

**FIGURE 2:**
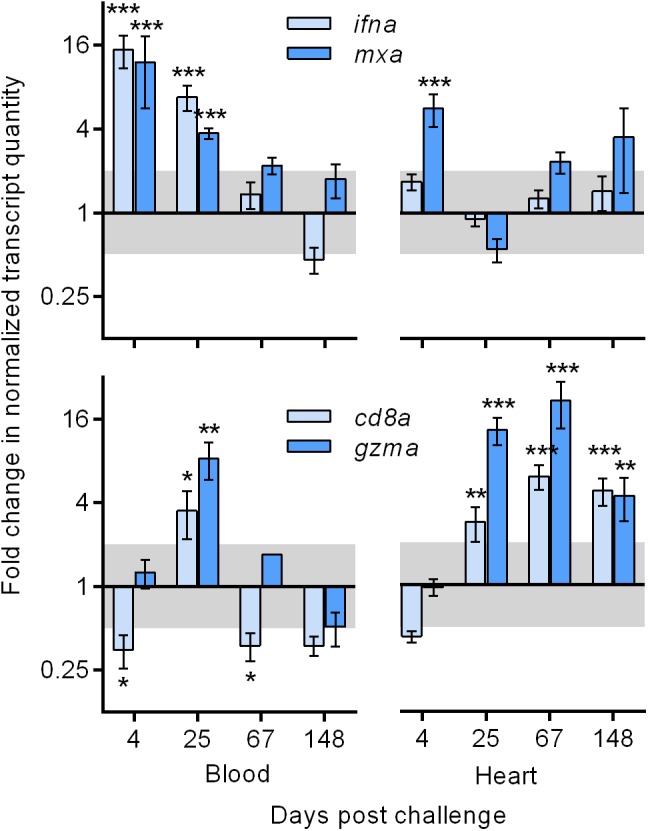
Systemic (blood) and heart associated transcriptional responses to PRV infection by Atlantic salmon. Mean (± s.e.m.; *n* = 8 per time point) fold change of transcripts associated with innate cellular recognition of virus (*ifna* and *mxa*) and adaptive cytotoxic T-cell directed killing (*cd8a* and *gzma*) in PRV challenged fish relative to time-matched BC fish normalized to β-actin. Significant (^∗^*p* < 0.05, ^∗∗^*p* < 0.01, ^∗∗∗^*p* < 0.001) changes of normalized relative quantity are indicated relative to BC. Shaded area indicates a less than twofold change unlikely to be biologically relevant. *ifna* – type 1 interferon gene; *mxa*, myxovirus resistance gene; *cd8a*, cluster of differentiation – 8 gene; *gzma*, granzyme-A gene.

PRV-infected salmon generated innate immune responses in the blood during initial and peak infection that contrasted the adaptive immune responses seen in heart tissues during the persistent infection phase. Expression of nine immune related genes was specifically targeted in this study. Transcription of genes encoding type-I interferon (*ifna*) and Myxovirus resistance (*mxa*) – two well characterized salmon genes involved in innate cellular recognition and defense against virus for nearly all cell types – were up-regulated by a mean of 12–15 fold early after PRV entry (4 dpc) and by a mean of 4–7 fold during peak viremia (25 dpc; Figure 2). This blood expression pattern was contrasted in heart tissues where only *mxa* significantly increased (mean sixfold) early after PRV entry. However, expression for the cluster of differentiation-8 receptor (*cd8a*) and granzyme-A (*gzma*) – two transcripts exclusively associated with adaptive immune cytotoxic T-cells and involved in intracellular antigen recognition and cell mediated killing, respectively – were significantly increased (mean of 4–20 fold) in heart tissues of PRV infected fish during the persistent phase of infection. This expression pattern was not reciprocated in blood cells in response to persistent PRV, which had generally low expression that was only significantly up-regulated (mean 4–8 fold) at 25 dpc during peak PRV viremia (Figure 2).

To further explore the putative involvement of other immune cells in PRV infected heart tissues, transcriptional gene expression of gamma interferon (*ifng*; expressed by natural killer cells, CD8+ T-cells and CD4+ T-cells) cluster of differentiation-4 receptor (*CD4*; expressed by CD4+ T-cells), interleukin-10 (*il10*; expressed by monocytes, mast cells, and B-cells), interleukin-12 (*il12*; expressed by dendritic cells and macrophages), and interleukin-18 (*il18*; expressed by macrophages) revealed that only *ifng* was significantly stimulated in response to PRV in heart tissues (Supplementary Figure 4). This suggests that CD8+ T-cells were likely the only immune cells involved in the recognition of PRV in this instance. Other transcriptional isoforms of interferon (i.e., *ifnb*, *ifnc*, and *ifnd*) have not shown responsiveness to this Pacific-Canada isolation of PRV in blood, purified erythrocyte, or heart tissues (MPP, unpublished data) and thus were not evaluated in this study.

### Respiratory Performance of PRV-Infected Salmon

#### Erythrocyte Oxygen Carrying Capability

**FIGURE 3:**
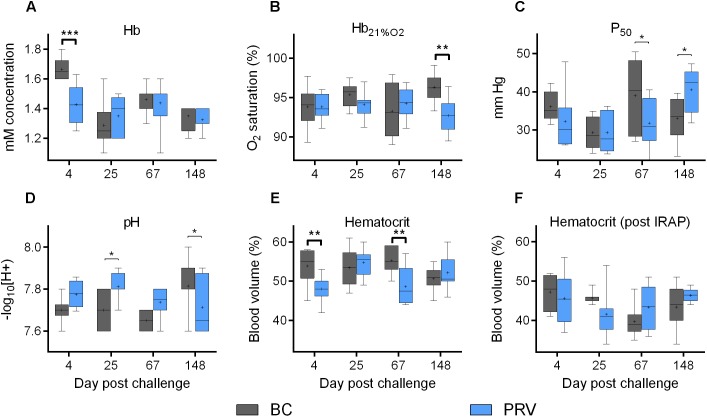
Erythrocyte oxygen transport capabilities of PRV infected Atlantic salmon. Bar and whisker plots (bar = 25–75 percentile; whiskers = min–max; line = median; plus = mean; *n* = 8) of five blood associated indices measured at 4 discrete sampling events. Significant difference (^∗^*p* < 0.05; ^∗∗^*p* < 0.01; ^∗∗∗^*p* < 0.001) in mean value between blood control injected (BC) and PRV injected (PRV) treatment groups are indicated at each time point as determined by uncorrected Fisher’s LSD comparison tests. Significance (*p* < 0.05) following family-wise *p*-value multiplicity adjustment (Bonferroni, Sidak, Holm–Sidak tests; α = 0.05) is indicated in bold. **(A–E)** Data collected from fish that did not undergo IRAP. **(F)** Data collected from fish immediately after IRAP. Hb, hemoglobin; Hb_21%O2_, Hb saturation at 21% oxygen; P_50_, partial pressure at 50% oxygen saturation.

The ability for erythrocytes to carry oxygen in Atlantic salmon was not impaired by PRV infection at any time point in this study despite a sustained, high-load PRV viremia, even when inclusion bodies were microscopically visible. Mean hematocrit (>40%) remained well above a previously estimated threshold of functional anemia (25%) in salmon (Gallaugher et al., 1995) throughout the 21-week experiment. While hematocrit of the BC- and PRV-treatment groups were always similar when sampled after respirometry, both hematocrit and Hb concentration were transitorily lower by about 7% for the PRV-treatment group at 4 dpc (Figure 3). At 67 dpc, hematocrit but not Hb concentration was again transitorily lower. Importantly, the BC injection significantly influenced hematocrit relative to SC at 4, 25, and 67 dpc (Supplementary Figure 5), suggesting that components of the foreign blood homogenate used for viral delivery in this study elicited its own minor physiological changes. Specific to PRV, neither Hb oxygen binding potential as measured by partial pressure required to achieve 50% saturation (P_50_) nor blood pH was affected by PRV at any infection stage as determined by two-way ANOVA or multiple comparison tests that control family-wise type-I error (Bonferroni, Sidak, Holm–Sidak; *p*-values provided in Zhang et al., 2019). Hemoglobin saturation with 21% oxygen (Hb_21%O2_) was significantly but only slightly lower in PRV-infected salmon at 148 dpc (Figure 3).

#### Respiratory Capacity and Capabilities

**FIGURE 4:**
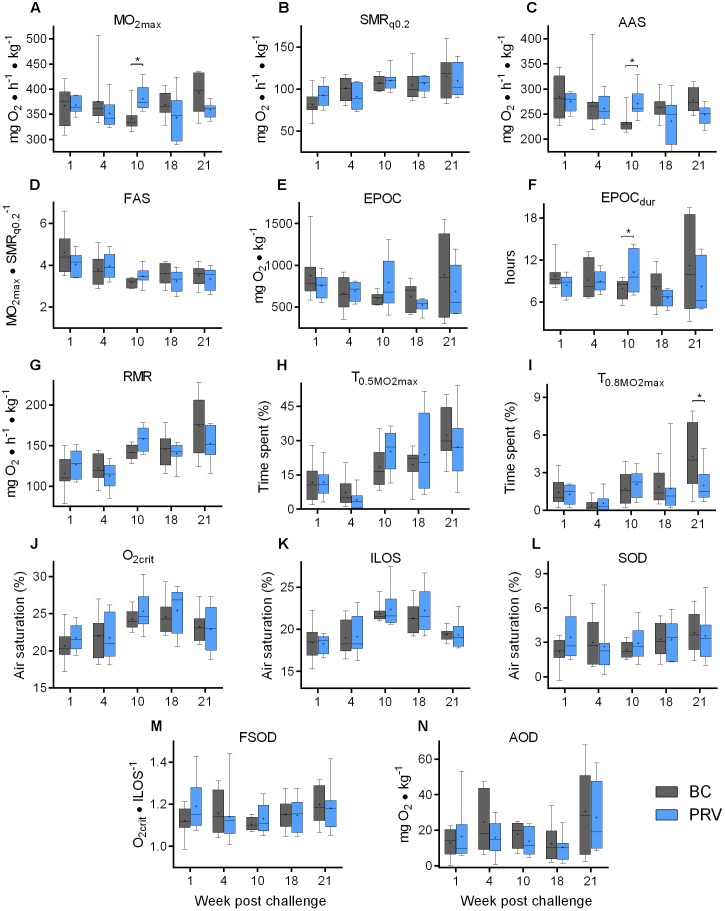
Respiratory performance of PRV injected Atlantic salmon. Bar and whisker plots (bar = 25–75 percentile; whiskers = min–max; line = median; plus = mean; *n* = 8) of respiratory indices measured at 5 discrete sampling events. Significant difference (^∗^*p* < 0.05) in mean value between blood control injected (BC) and PRV injected (PRV) treatment groups are indicated at each time point as determined by uncorrected Fisher’s LSD comparison tests. No comparisons were significantly different following family-wise *p*-value multiplicity adjustment (Bonferroni, Sidak, or Holm–Sidak tests; α = 0.05). Data at 21 wpc is repeated measure of fish assessed at 18 wpc. **(A)** 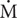
O_2max_, maximum metabolic rate; **(B)** SMR_q0.2_, standard metabolic rate (0.2-quartile method); **(C)** AAS, absolute aerobic scope; FAS, factorial aerobic scope; **(E)** EPOC, excess post-exercise oxygen consumption; **(F)** EPOC_dur_, EPOC duration; **(G)** RMR, routine metabolic rate; **(H)** T_0.5
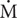
O_2max__, time spent above 50% of 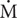
O_2max_; **(I)** T_0.5
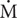
O_2max__, time spent above 80% 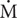
O_2max_; **(J)** O_2crit_, critical oxygen level; **(K)** ILOS, incipient lethal oxygen saturation; **(L)** SOD, scope of oxygen deficit; **(M)** FSOD, factorial SOD; **(N)** AOD, accumulated oxygen deficit.

Piscine orthoreovirus viremia had no major or sustained effects on the respiratory indices measured in Atlantic salmon during any phase of the infection process. Neither two-way ANOVA, Bonferroni, Sidak, nor Holm-Sidak comparisons identified significant (*p* < 0.05) differences between PRV and BC treatment groups for any of the respiratory indices tested at any time point during the study (Figure 4; Zhang et al., 2019). Fisher’s LSD, which is the only test used in this study that does not control for family-wise type-I error, identified four small significant differences. At week 10, PRV-infected salmon had a 12 ± 3% (mean ± s.e.m.) higher 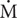
O_2max_, a 16 ± 4% higher AAS and a 30 ± 13% higher EPOC_dur_, while at week 21, PRV-infected salmon had a 19 ± 22% lower T_0.8
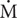
O2max_ (Figure 4). One potential interpretation of these results is that PRV subtly improved aerobic capacities during the persistent phase of infection that necessitated a longer recovery period following exhaustion. A more probable explanation, however, is that these changes are contained within normal biological variability and that significance assigned in these instances (4 per 70 total comparisons; 5.7%) represents type I family-wise error uncontrolled by Fisher’s LSD (Wright, 1992). This is supported by the lack of significance identified by two-way ANOVA in comparing treatments across all four non-repeated measures sampling events (*p* > 0.26 for each index). This is also supported by the fact that BC injection alone caused significant (*p* < 0.05 family-wise error adjusted) contributions to aerobic capabilities (23 ± 4% increase in SMR; 24 ± 2% decrease FAS) and exhaustion recovery (26 ± 5% decrease in EPOC_dur_) at 10 wpc relative to SC injection (Supplementary Figure 6), and that time also had a significant effect on SMR, FAS, EPOC, EPOC_dur_, RMR, T_0.5
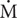
O2max_, T_0.8
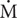
O2max_ and ILOS as determined by two-way ANOVA (Zhang et al., 2019) – observations that suggest physiological plasticity. Indeed, principle component analysis suggested that, independent of treatment, time in association with length and fish condition were the predictor variables contributing to the greatest variance in respiratory performance in this study (Supplementary Figure 7). Subsequent targeted consideration of time in relation to respiratory indices in the SC group identified significant (*p* < 0.05) temporal effects on aerobic activity, exercise recovery, routine activity, agitation level, and anaerobic performance (Supplementary Figure 8). Specifically, baseline aerobic activity (SMR) increased 40 ± 6% over 18 weeks of non-repeated measures sampling and was accompanied by a 55 ± 8% increase in routine activity level (RMR), 353 ± 88% increase in mild agitation (T_0.5
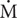
O2max_), 33 ± 7% increase in the rate of recovery from exhaustion (both EPOC and EPOC_dur_) and a 20–30% decrease in tolerance to hypoxia (20 ± 2% increase O_2crit_; 29 ± 4% increase ILOS).

Furthermore, we found that tolerance to hypoxia at 21 wpc was improved after the severe acute hypoxia challenge at 18 wpc, which suggests that the hypoxia tolerance in Atlantic salmon may be a phenotypically plastic trait. Fish in all treatment groups had improved ILOS (mean decrease 11 ± 1%) during their second acute hypoxia exposure which was administered 3 weeks after their first (Supplementary Figure 9). However, no respiratory indices were statistically different between SC, BC, and PRV treatment groups at 21wpc following *p*-value multiple comparison adjustment (Figure 4 and Supplementary Figure 6). Lastly, regardless of whether BC treated Atlantic salmon did or did not contain mild heart inflammation, there was no significant impairment to any of the physiological performance measurements. In fact, the cumulative variability of BC treated fish was almost always encompassed within the variability observed for the SC group. When significant differences in physiological parameters were observed between BC and SC fish, it was not temporally aligned with the highest prevalence of heart inflammation (10 wpc vs. 21 WPC), further suggesting the minor heart inflammation in BC treatment group had no effect on respiratory physiology (Supplementary Figure 10).

## Discussion

As an essential life-support system, cardiorespiratory function can be differentially fine-tuned to the swimming needs of specific populations of wild Pacific salmon (Eliason et al., 2011). Thus, the consequences of systematic infections that harm erythrocyte and cardiac functions should be readily evident from impaired respiratory performance. We discovered, however, that high-load PRV viremia in Atlantic salmon did not affect oxygen affinity and carrying capacity of erythrocytes, nor could sustained or appreciable differences be identified in 14 indices of respiratory capabilities despite transient cellular activation of antiviral response pathways. The lack of functional harm to Atlantic salmon infected with PRV highlights that, in a current era of unprecedented virus discovery, infection does not necessarily imply bodily harm and viral load is not always a suitable predictor of disease within a host organism. Whether other subtypes of PRV, such as the PRV1 associated with HSMI in Norway or PRV2 associated with anemia in Japan, harms cardiorespiratory function as has been previously suggested (Olsen et al., 2015; Takano et al., 2016; Lund et al., 2017) still needs further experimental testing. It is also unknown if variable temperatures may affect PRV associated disease or how this may impact host physiological responses if or when disease occurs.

The high-load viremia and temporal infection dynamics resulting from injection of a PRV-loaded blood homogenate in this study closely mirrored previous challenge trials with Atlantic salmon in Pacific Canada (Garver et al., 2016; Polinski et al., 2019). However, here we increased the inoculating dose approximately 10-fold compared to previous work and harvested the virus during peak replication to maximize the PRV load and infection intensity. Even so, peak systemic PRV loads and temporal phases of infection remained nearly identical to previous studies, indicating that a maximum systemic PRV carrying capacity was likely reached at approximately 10–100 billion genomic copies per mL blood – a capacity that is equal to, if not higher, than loads generated in natural or aquaculture systems (Lovoll et al., 2012; Garseth et al., 2013; Marty et al., 2015). Therefore, our laboratory study simulated a relevant ‘worst-case’ model for assessing physiological harm associated with PRV load intensity.

Given that PRV specifically targets the heart and erythrocytes of Atlantic salmon, our hypothesis was comprehensively tested by assessing: (i) oxygen binding properties of erythrocytes; (ii) respiratory capabilities in normoxia; (iii) respiratory capabilities in hypoxia; and (iv) respiratory capabilities after an acute hypoxic stress. Transient cytoplasmic inclusions of erythrocytes are a common attribute of acute systemic PRV infection and indicative of viral replication (Finstad et al., 2014; Wessel et al., 2015; Polinski et al., 2019). Here, we provide evidence that oxygen binding properties of erythrocytes are unaffected by these erythrocyte inclusions and correspondingly high PRV transcriptional loads even with the erythrocytes having transient innate immune responses as observed previously (Polinski et al., 2019). Importantly, these innate responses cannot be energetically expensive because neither SMR, nor viral replication, nor erythrocyte function was affected. Thus, we conclude that the PRV replication process within erythrocytes was likely commensal to normal cellular functioning in this instance.

While the primary site of PRV replication is presumed to be within erythrocytes (Finstad et al., 2014; Garver et al., 2016; Polinski et al., 2019), cardiomyocytes are a site for secondary infection linked to the development of HSMI in Atlantic salmon. Indeed, both innate (interferon) and adaptive (cytotoxic T-cell) immune responses have been implicated in this cardiac response (Mikalsen et al., 2012). Here, we confirmed increased transcription of markers for cytotoxic T-cells (*cd8a*) and their directed killing of target cells (*grzm*) in PRV-infected heart tissues, even in the absence of notable heart inflammation. However, the heart initiated only minor transcription of innate interferon responses to virus (*ifna* and *mxa*), which suggests that (i) innate immune recognition of PRV via pattern recognition receptors (PRRs) is unlikely involved in the initiation of PRV-associated inflammation, and (ii) major histocompatibility complex-1 (MHC-1) presentation of PRV antigen to mature cytotoxic T-cells is most probably the initiator for the inflammatory heart lesions associated with PRV. This is supported by the previous identification of significant T-cell involvement (Yousaf et al., 2012, 2013), specifically CD8 cytotoxic T-cells (Mikalsen et al., 2012), during HSMI. Our study also suggests that the other immune cells putatively involved during HSMI identified in previous studies (e.g., B-cells, macrophages and CD4+ helper T-cells) may be secondarily activated as a result of severe inflammation and necrosis rather than in response to PRV directly; although virus genotypic factors may also play a role. Why erythrocytes are not similarly targeted for cytotoxic T-cell mediated killing via MHC-1 presentation in the blood is currently unclear.

The North American genotype of PRV1 in our study produced mild and occasionally moderate heart inflammation in a Pacific domesticated Mowi-McConnell strain of Atlantic salmon using an apparently maximum virulence model system (Garver et al., 2016; Polinski et al., 2019). This was clearly not HSMI, as the severity of inflammation was generally indistinguishable from control fish. It was certainly not associated with compromised heart function; otherwise the respiratory capabilities of PRV-infected salmon would not have been as robust as those of controls. By comparison, a Norwegian genotype of PRV1, which caused moderate heart lesions (classified as HSMI) in a cohabitation model system, produced similar PRV loads and yielded transient impairments to some but not all cardiorespiratory functions (Lund et al., 2017). The juxtaposition of these regionally divergent findings supports three important conclusions:

(i) The Atlantic salmon heart is functionally resilient to mild (present study) and perhaps moderate (Lund et al., 2017) inflammation; whether severe inflammation affects cardiac performance has yet to be tested.
(ii) Neither the presence, nor a high systemic load of PRV is a useful proxy for predicting disease in salmon. While particularly evident in western North America (and possibly elsewhere), it also appears true in Norway given that many PRV-infected salmon farms do not develop HSMI (Lovoll et al., 2012) and systemic PRV loads in Norway challenge trials do not always correlate with heart inflammation severity (Lund et al., 2017; Wessel et al., 2017).
(iii) There is regional disparity in PRV’s ability to cause respiratory harm. Identifying how much of this regional variability is dependent on viral genotype, host genotype, or environmental factors requires further investigation.

Despite the lack of sustained and major changes in the IRAP indices as a result of severe viremia associated with the PRV injection, many of the IRAP indices did change within the SC treatment over 18 weeks, as did some of the BC and PRV treatment groups. Whether these changes (e.g., increased SMR and RMR along with decreased EPOC and EPOC_dur_) were a result of time in seawater, greater exploratory behaviors, progressively reducing the fish population in the tank, or related to growth is unclear. However, they point to physiological plasticity, as did the changes seen following the severe hypoxic challenge (e.g., reduced ILOS) evaluated at 21 wpc. The present findings also revealed that a saline injection is not an adequate control for injecting PRV-loaded blood homogenate into Atlantic salmon because significant differences were seen been SC and BC treatments for a few respiratory indices (e.g., increased transient SMR and reduced FAS and EPOC_dur_). Lastly, although the respiratory responses to the extreme hypoxic exposure were notable, they must be applied with great caution since such a severe exposure is highly unlikely in either a wild or farmed salmon setting and would have led to mortality had we not actively revived fish after they had lost their righting reflex.

## Conclusion

This study provides strong empirical evidence that a host with an extremely high-load PRV infection can sustain its respiratory functions. It also supports previous laboratory investigations which have found PRV to be orphaned from establishing a physiologically relevant disease state in salmon of western North America and provides robust evidence to dispel the concern that respiratory performance is compromised by PRV in the absence of overt disease. Thus, the global concern about the negative impact of this virus to salmon should be reassessed. Indeed, while several studies have associated PRV with diseased salmon in Pacific Canada (Di Cicco et al., 2017, 2018), current experimental evidence does not suggest that PRV is the only factor for initiating these disease conditions (Garver et al., 2015, 2016; Polinski et al., 2016, 2019) which may have a more complex etiology. Regardless, PRV emerges as an atypical example of a virus with putative pathogenic properties because the total quantity of virus within an infected organism appears to have little to no bearing on whether the organism becomes diseased and experiences physiological harm. In contrast, this study supports the hypothesis that host recognition rather than direct PRV virulence is likely a key and variable factor in shifting this putative commensal relationship into a disease state. This becomes an important paradigm to consider in assessing the disease causing potential of hitherto uncharacterized animal viruses as they are discovered.

## Data Availability Statement

The data collected for this study can be found in the FigShare digital repository (Zhang et al., 2019).

## Author Contributions

YZ conducted the respiratory assessments, preformed the analyses on respiratory data, and collaborated in drafting the manuscript. MP aided in respiratory assessments, conducted the tissue sampling, analyzed the molecular data, and drafted the manuscript. PM performed the erythrocyte oxygen affinity and carrying capacity assessments. AF and KG conceived the study. MP, CB, AF, and KG designed the study. All authors read, contributed to, and approved the final manuscript.

## Conflict of Interest Statement

The authors declare that the research was conducted in the absence of any commercial or financial relationships that could be construed as a potential conflict of interest.

## Footnotes

1 marineharvest.com
