# Corrigendum: High-Load Reovirus Infections Do Not Imply Physiological Impairment in Salmon

**DOI:** 10.3389/fphys.2019.01354

**Published:** 2019-10-29

**Authors:** Yangfan Zhang, Mark P. Polinski, Phillip R. Morrison, Colin J. Brauner, Anthony P. Farrell, Kyle A. Garver

**Affiliations:** ^1^Faculty of Land and Food Systems, The University of British Columbia, Vancouver, BC, Canada; ^2^Aquatic Diagnostics and Genomics Division, Pacific Biological Station, Fisheries and Oceans Canada, Nanaimo, BC, Canada; ^3^Department of Zoology, The University of British Columbia, Vancouver, BC, Canada

**Keywords:** piscine orthoreovirus, salmon, cardiorespiratory performance, heart inflammation, viremia, nucleated erythrocytes

There is an error in the **Funding** statement. We neglected to include the funding number for “Aquaculture Collaborative Research and Development Program within Fisheries and Oceans Canada.” The funding number should be “16-1-P-03.”

Furthermore, we neglected to include that the funder “Aquaculture Collaborative Research and Development Program within Fisheries and Oceans Canada, 16-1-P-03” to KG involved collaborative support from “The British Columbia Salmon Farmers Association.”

A correction has been made to the **Funding** statement:

“This study was supported by a grant from the Aquaculture Collaborative Research and Development Program within Fisheries and Oceans Canada, 16-1-P-03 awarded to KG. Collaborative support in this instance was provided by the British Columbia Salmon Farmers Association. The British Columbia Salmon Farmers Association (or members therein) did not participate in the experimental study design, data collection and analysis, preparation of the manuscript, decision to publish, and hold no intellectual property rights associated with data or procedures developed in this study.”

The authors apologize for this error and state that this does not change the scientific conclusions of the article in any way. The original article has been updated.

