# Supplementary material for: High-Load Reovirus Infections Do Not Imply Physiological Impairment in Salmon
Source: Front Physiol. 2019 Mar 13;10:114. doi: 10.3389/fphys.2019.00114 (PMC6425399; doi:10.3389/fphys.2019.00114)
Supplement: Supplementary file 1 [file Data_Sheet_1.pdf]

Supplementary Material

High-load reovirus infections do not imply physiological impairment in salmon

Yangfan Zhang[1†], Mark P. Polinski[2†*], Phillip R. Morrison[3], Colin J. Brauner[3], Anthony P. Farrell[1,3], Kyle A. Garver[2*]

[1] Faculty of Land and Food Systems, The University of British Columbia, Vancouver, BC, Canada, [2] Aquatic Diagnostics and Genomics Division, Pacific Biological Station, Fisheries and Oceans Canada, Nanaimo, BC, Canada, [3] Department of Zoology, The University of British Columbia, Vancouver, BC, Canada

*** Correspondence:** Mark P. Polinski: Mark.Polinski@dfo-mpo.gc.ca
Kyle A. Garver: Kyle.Garver@dfo-mpo.gc.ca

[†] These authors have contributed equally to this work

Article citation:
Zhang Y, Polinski MP, Morrison PR, Brauner CJ, Farrell AP and Garver KA (2019) High-Load Reovirus Infections Do Not Imply Physiological Impairment in Salmon. *Front. Physiol.* 10:114. doi: 10.3389/fphys.2019.00114

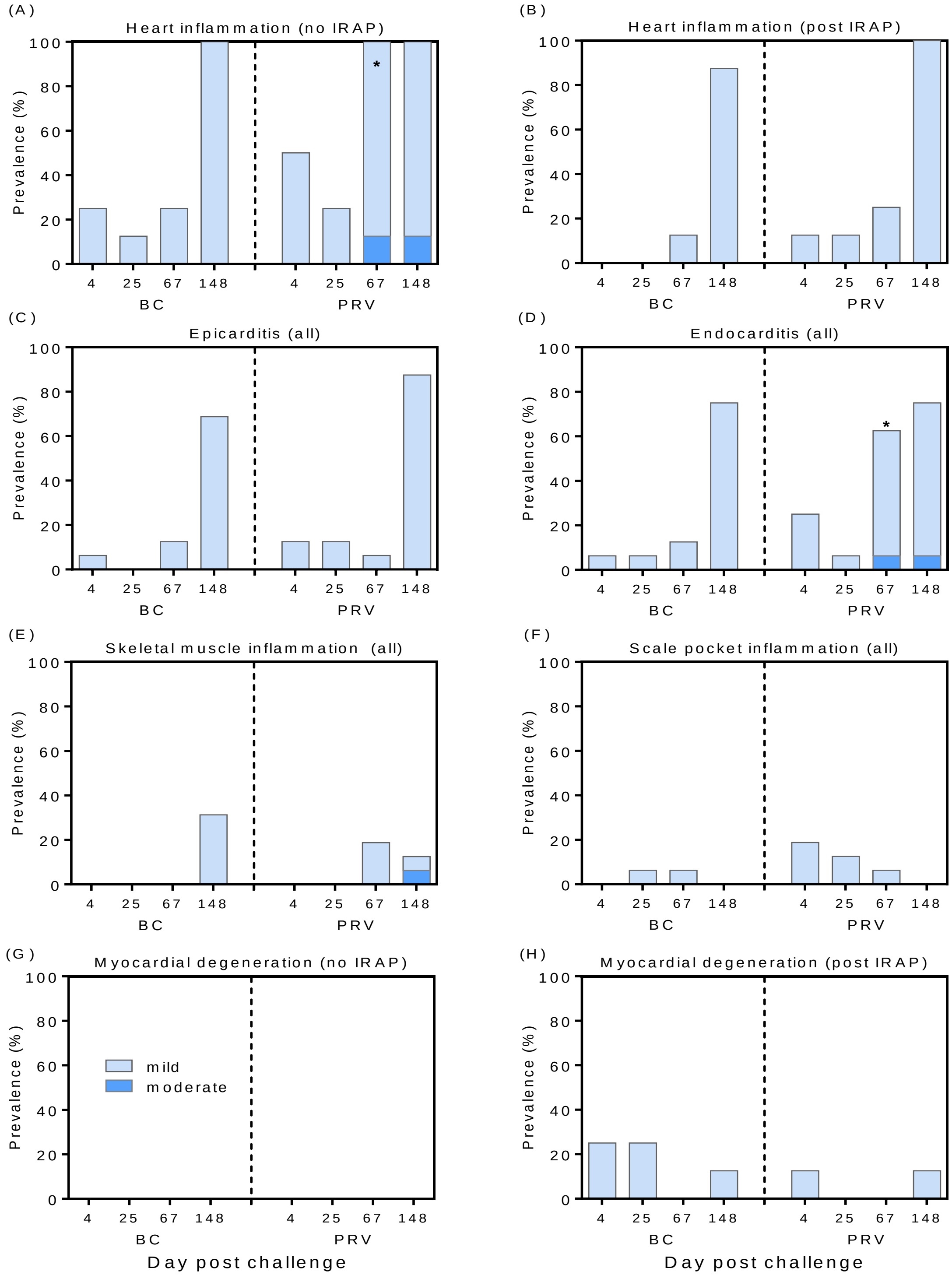

Supplementary Figure 1. Prevalence and severity of heart and skeletal muscle lesions. Heart inflammation in blood control (BC) or PRV infected (PRV) Atlantic salmon **(A)** directly from experimental holding tanks (no IRAP; n=8 per time point) or **(B)** immediately following IRAP assessment (post IRAP; n=8 per time point). **(C)** Epicarditis, **(D)** endocarditis, **(E)** skeletal muscle inflammation and **(F)** scale pocket inflammation in combined IRAP and non-IRAP assessed fish (n=16 per time point). Myocardial degeneration of skeletal muscle in fish sampled **(G)** directly from experimental holding tanks (no IRAP; n=8 per time point) or **(H)** immediately following IRAP assessment (post IRAP; n=8 per time point). (*) Indicates a significant ($p < 0.05$) increase in median pathology score in PRV challenged groups relative to time matched BC by Mann-Whitney U test.

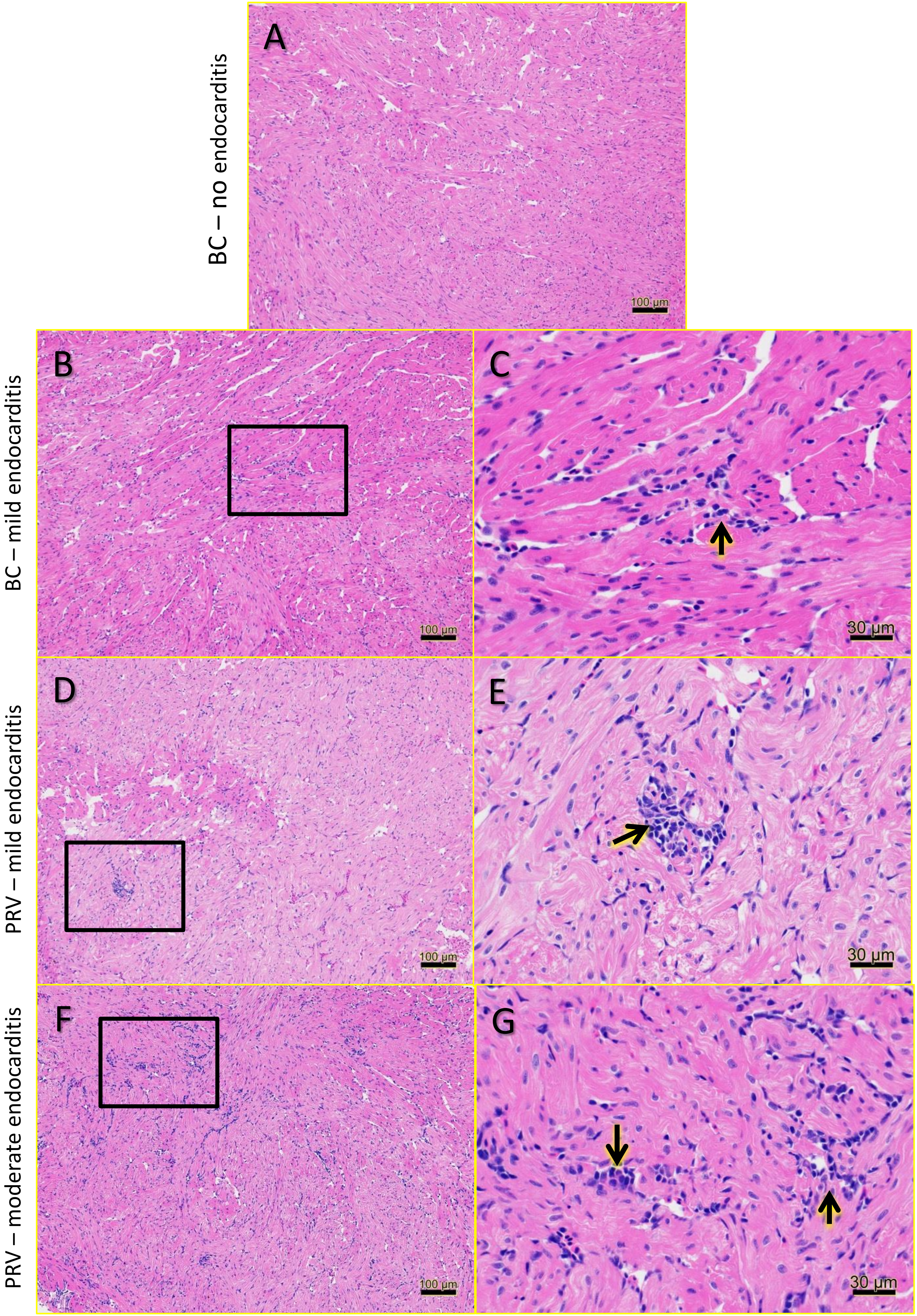

BC – no endocarditis
BC – mild endocarditis
PRV – mild endocarditis
PRV – moderate endocarditis
A
B
C
D
E
F
G
100 µm
30 µm

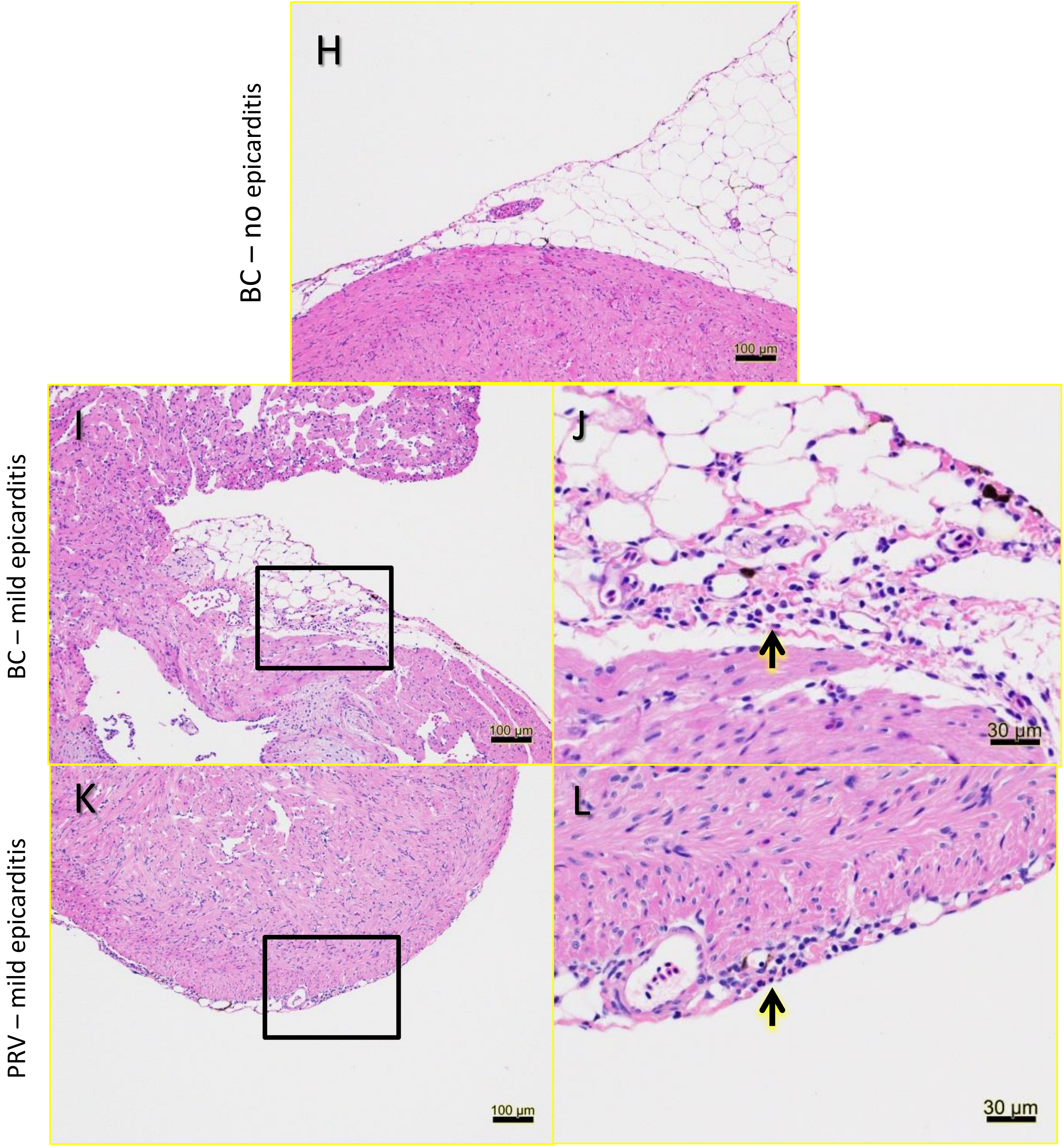

Supplementary Figure 2. Histopathology micrographs of heart inflammation. **(A)** Example of normal endocardium (score 0) from BC fish #23; **(B-C)** example of endocardium with mild (score 1) lymphohistiocytic endocarditis in BC fish #74; **(D-E)** example of endocardium with mild (score 1) lymphohistiocytic endocarditis in PRV fish #150; **(F-G)** example of endocardium with moderate (score 2) lymphohistiocytic endocarditis in PRV fish #145; **(H)** example of normal epicardium (score 0) from BC fish #23; **(I-J)** example of epicardium with mild (score 1) lymphohistiocytic epicarditis in BC fish #138; **(K-L)** example of epicardium with mild (score 1) lymphohistiocytic epicarditis in PRV fish #158. Boxed area in left panel images outlines corresponding images in right panel at higher magnification. Arrows in right panel images indicate areas of inflammation.

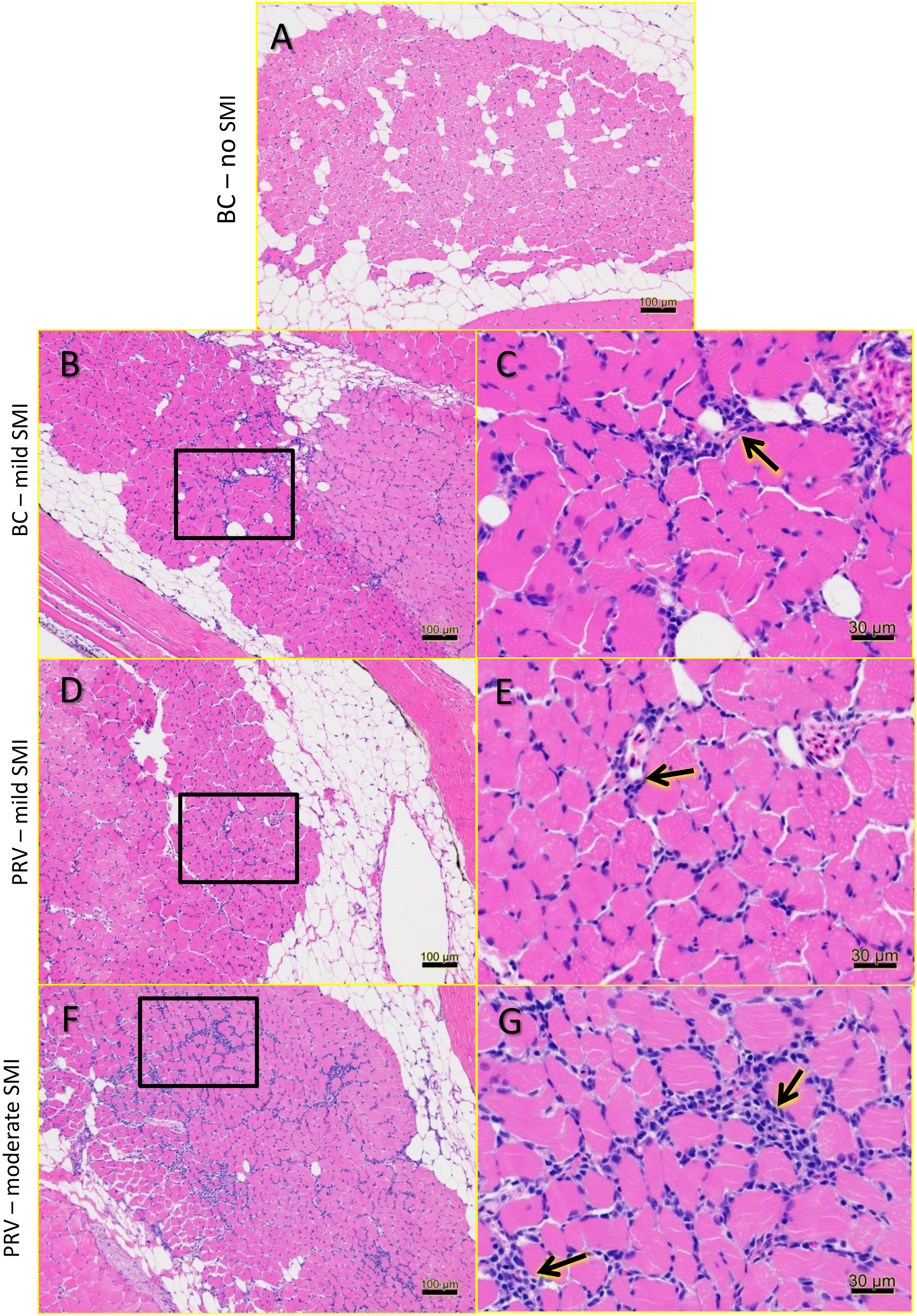
BC – no SMI
BC – mild SMI
PRV – mild SMI
PRV – moderate SMI
A
B
C
D
E
F
G
100 μm
30 μm

Supplementary Figure 3. Histopathology micrographs of skeletal muscle inflammation. **(A)** Example of normal skeletal muscle (score 0) from BC fish #23; **(B-C)** example of skeletal muscle with mild (score 1) inflammation in BC fish #204; **(D-E)** example of skeletal muscle with mild (score 1) inflammation in PRV fish #220; **(F-G)** example of skeletal muscle with moderate (score 2) inflammation in PRV fish #228. Boxed area in left panel images outlines corresponding images in right panel at higher magnification. Arrows in right panel indicate areas of inflammation.

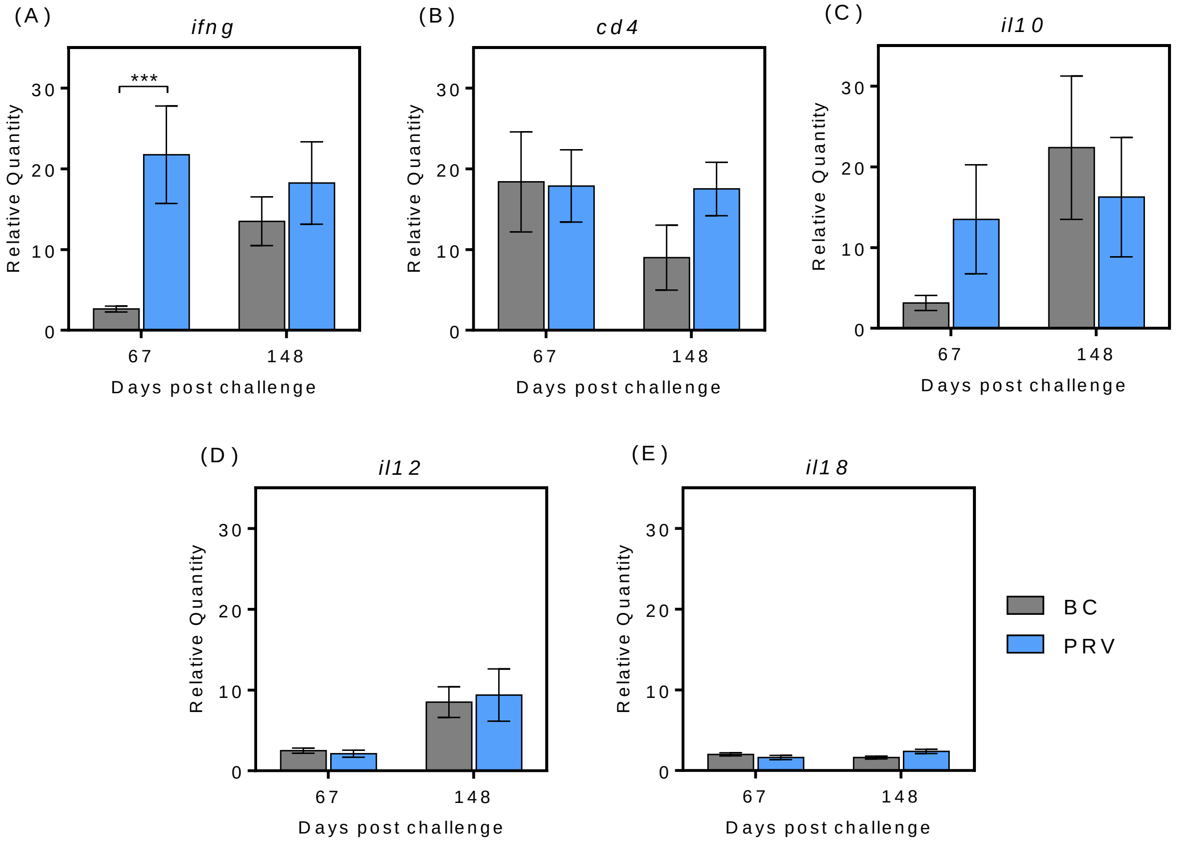

Supplementary Figure 4. Heart associated immune related transcriptional responses to PRV infection by Atlantic salmon during the persistent phase of infection associated with minor heart inflammation. Mean (± s.e.m.; n=8 per time point) relative quantity of transcripts associated with **(A)** natural killer cells/CD8+ T-cells/CD4+ T-cells (*ifng*), **(B)** CD4+ T-cells (*cd4*), **(C)** monocytes/mast cells/B-cells (*il10*), **(D)** dendritic cells/macrophages (*il12*), or **(E)** macrophages (*il18*) in PRV and time-matched BC treatment groups normalized to β-actin. Significant (* $p < 0.05$, ** $p < 0.01$, *** $p < 0.001$) changes of normalized relative quantity between treatment at each time point are indicated. *ifng* = interferon γ; *cd4* = cluster of differentiation-4; *il10* = interleukin-10; *il12* = interleukin-12; *il18* = interleukin-18.

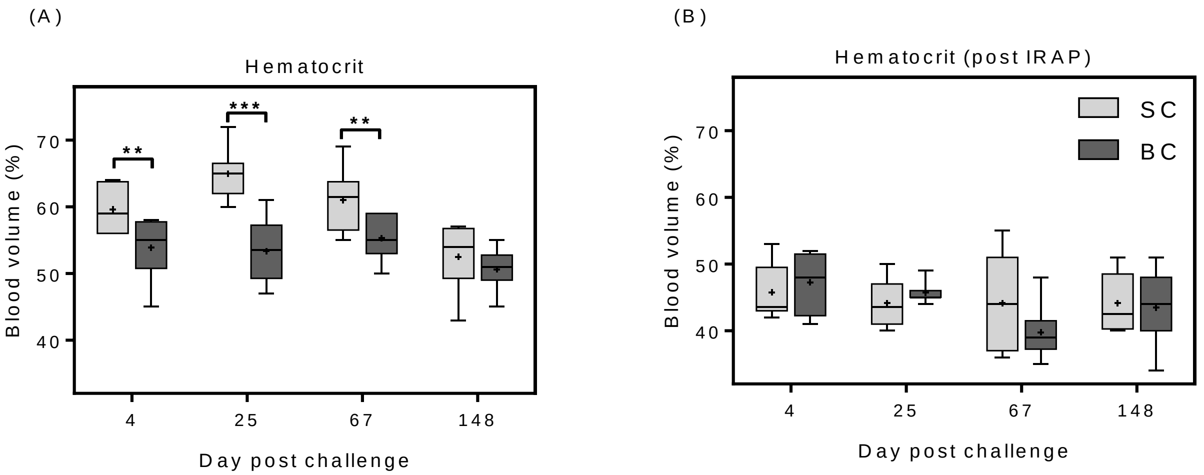

Supplementary Figure 5. Hematocrit of blood control inoculated Atlantic salmon. Bar and whisker plots (bar = 25-75 percentile; whiskers = min-max; line = median; plus = mean; n = 8) of hematocrit measured at 4 discrete sampling events. Significant difference (* $p < 0.05$; ** $p < 0.01$; *** $p < 0.001$) in mean value between saline injected (SC) and blood control injected (BC) treatment groups are indicated at each time point as determined by uncorrected Fisher's LSD comparison tests. Significance ($p < 0.05$) following family-wise p-value multiplicity adjustment (Bonferroni, Sidak, Holm-Sidak tests; $\alpha = 0.05$) is indicated in bold. **(A)** Data collected from fish that did not undergo IRAP. **(B)** Data collected from fish immediately after IRAP.

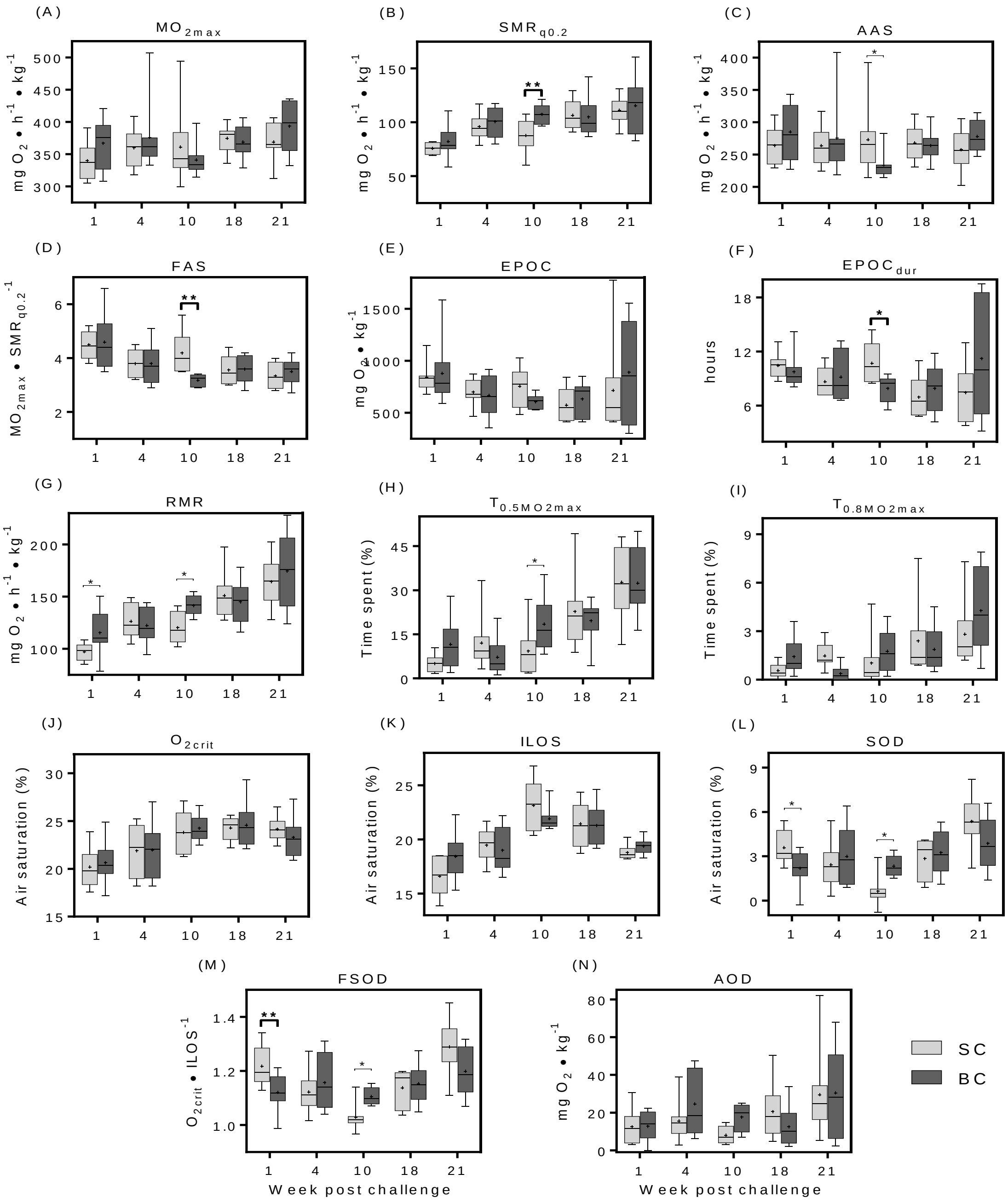

Supplementary Figure 6. Respiratory performance of blood control inoculated Atlantic salmon. Bar and whisker plots (bar = 25-75 percentile; whiskers = min-max; line = median; plus = mean; n = 8) of respiratory indices measured at 5 discrete sampling events. Significant difference (* $p < 0.05$; ** $p < 0.01$; *** $p < 0.001$) in mean value between saline injected (SC) and blood control injected (BC) treatment groups are indicated at each time point as determined by uncorrected Fisher's LSD comparison tests. Significant difference ($p < 0.05$) following family-wise p-value multiplicity adjustment (Bonferroni, Sidak, Holm-Sidak tests; $\alpha = 0.05$) is indicated in bold. Data at 21 wpc is repeated measure of fish assessed at 18 wpc. **(A)** $\dot{M}$O2max = maximum metabolic rate; **(B)** SMRq0.2 = standard metabolic rate (0.2-quartile method); **(C)** AAS = absolute aerobic scope; **(D)** FAS = factorial aerobic scope; **(E)** EPOC = excess post-exercise oxygen consumption; **(F)** EPOCdur = EPOC duration; **(G)** RMR = routine metabolic rate; **(H)** T0.5$\dot{M}$O2max = time spent above 50% of $\dot{M}$O2max; **(I)** T0.5$\dot{M}$O2max = time spent above 80% $\dot{M}$O2max; **(J)** O2crit = critical oxygen level, **(K)** ILOS = incipient lethal oxygen saturation; **(L)** SOD = scope of oxygen deficit; **(M)** FSOD = factorial SOD; **(N)** AOD = accumulated oxygen deficit.

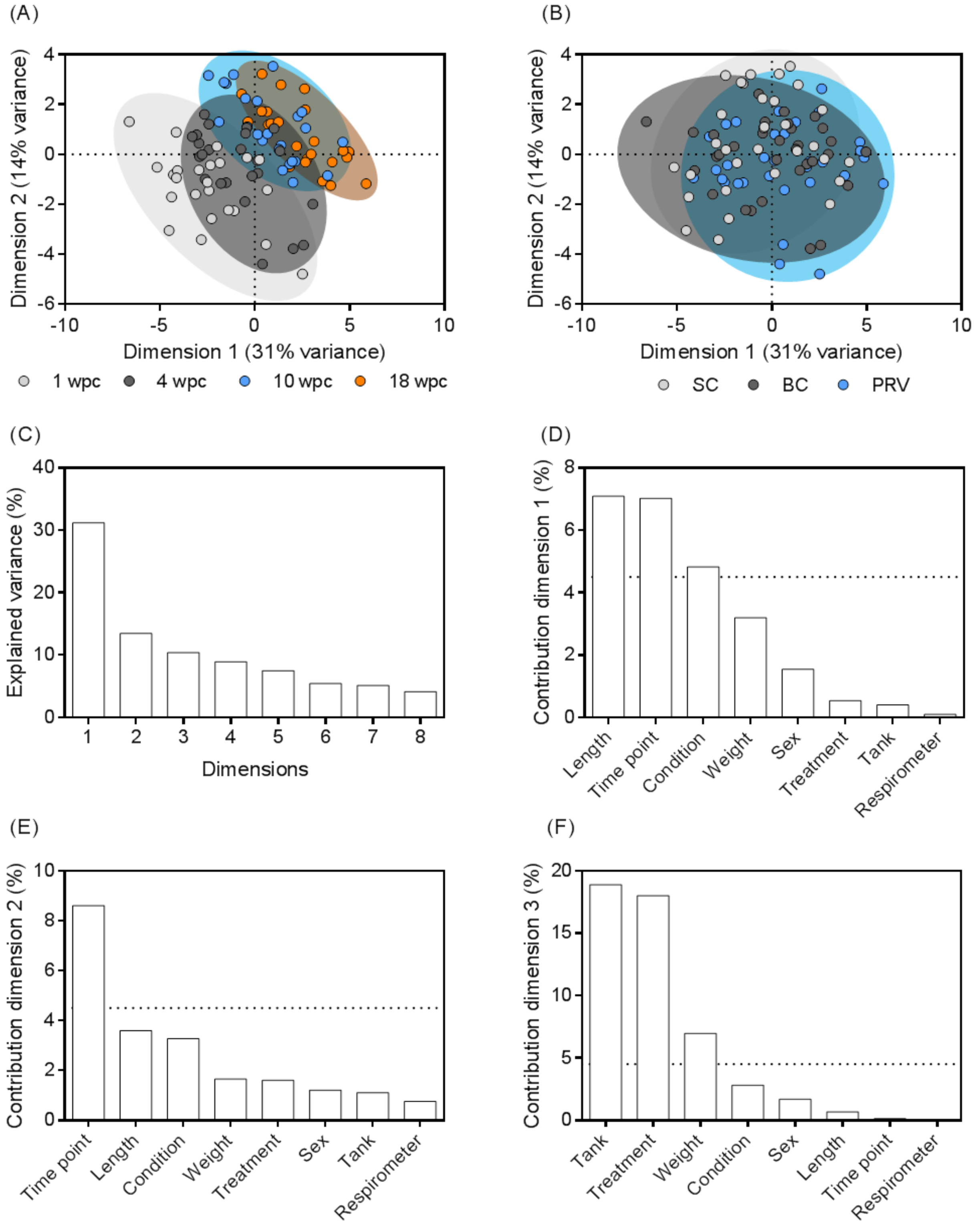

Supplementary Figure 7. Exploration of variance in IRAP associated data using principal component analysis. Component scores grouped by **(A)** time point or **(B)** treatment in reference to the two principle dimensions of variance. **(C)** The contribution to total variance associated with each of the eight principle dimensions. **(D-F)** Contributions of eight putative predictor variables to the total variance within the first three principle dimensions of variance. Dotted lines correspond to an expected (uniform) contribution.

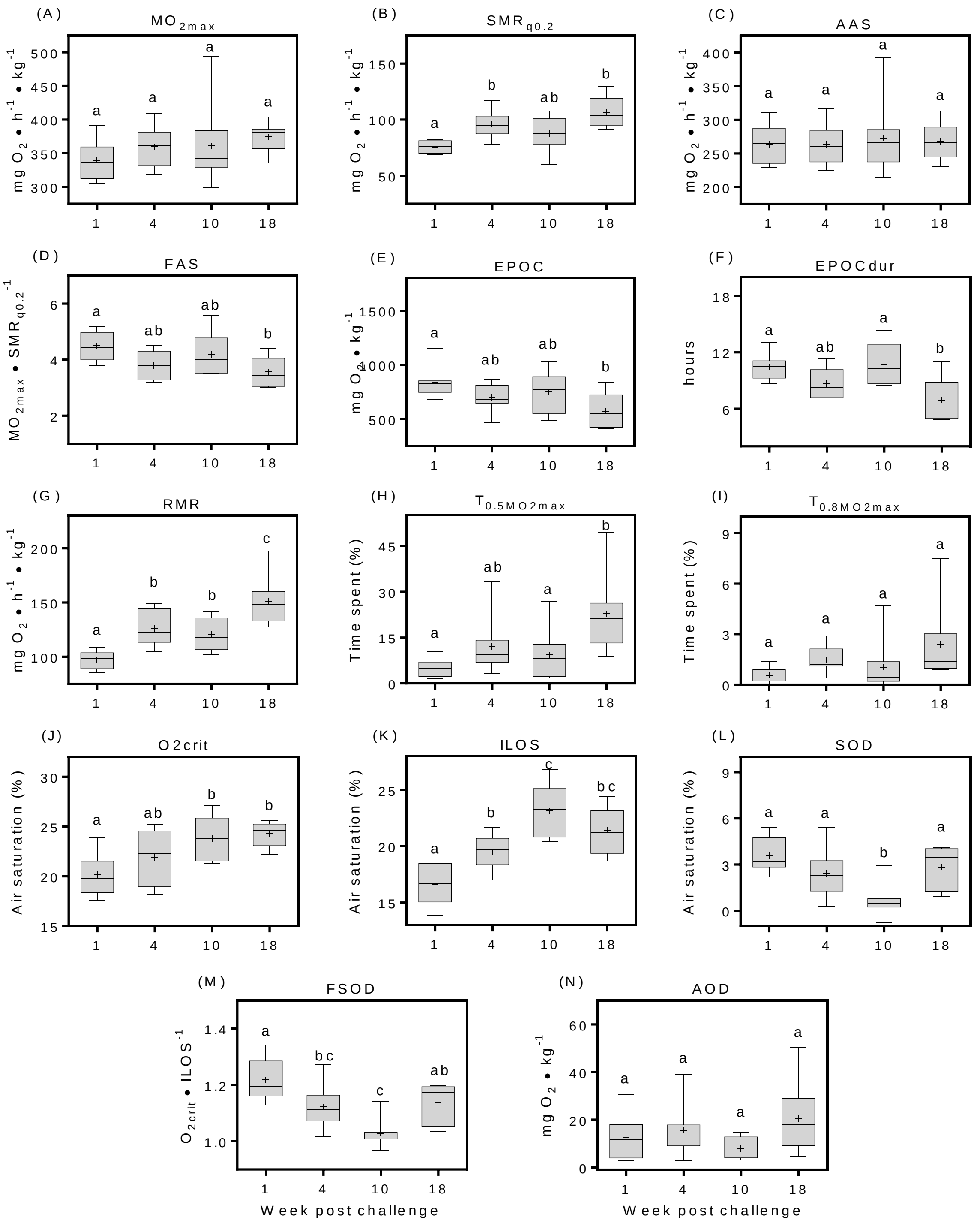

Supplementary Figure 8. Respiratory performance of saline control (SC) inoculated Atlantic salmon during 18 weeks of non-repeated measure sampling. Bar and whisker plots (bar = 25-75 percentile; whiskers = min-max; line = median; plus = mean; n = 8) of 14 respiratory indices. Letters denote comparable mean quantities at a significance threshold of $p < 0.05$ as determined by Tukey's HSD multiple comparison tests. **(A)** ṀO2max = maximum metabolic rate; **(B)** SMRq0.2 = standard metabolic rate (0.2-quartile method); **(C)** AAS = absolute aerobic scope; **(D)** FAS = factorial aerobic scope; **(E)** EPOC = excess post-exercise oxygen consumption; **(F)** EPOCdur = EPOC duration; **(G)** RMR = routine metabolic rate; **(H)** T0.5ṀO2max = time spent above 50% of ṀO2max; **(I)** T0.5ṀO2max = time spent above 80% ṀO2max; **(J)** O2crit = critical oxygen level, **(K)** ILOS = incipient lethal oxygen saturation; **(L)** SOD = scope of oxygen deficit; **(M)** FSOD = factorial SOD; **(N)** AOD = accumulated oxygen deficit.

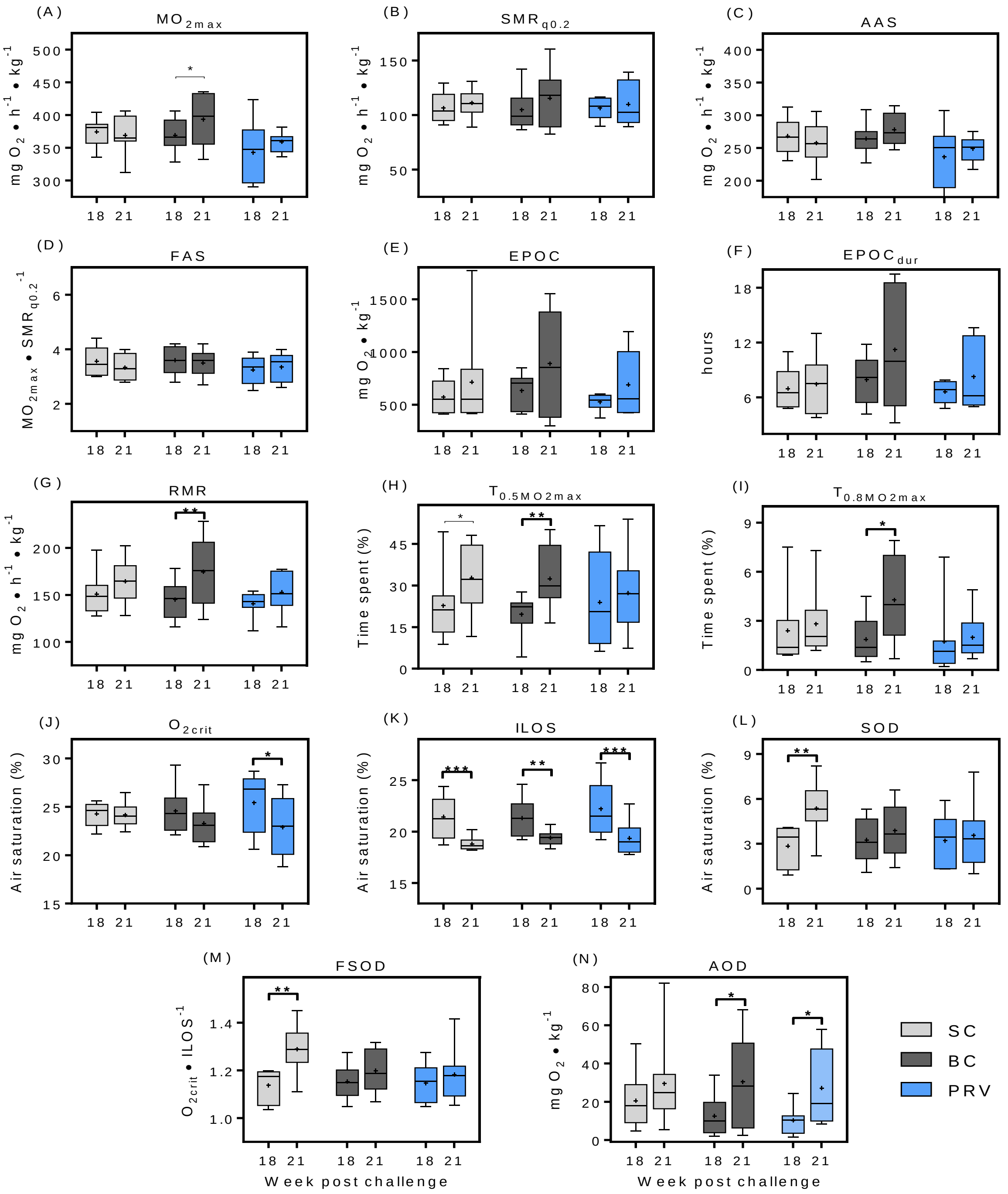

Supplementary Figure 9. Indication for phenotypic plasticity of Atlantic salmon to acute hypoxia challenge. Bar and whisker plots (bar = 25-75 percentile; whiskers = min-max; line = median; plus = mean; n =8) of 14 respiratory indices for fish exposed to an IRAP at 18 wpc and then reassessed at 21 wpc. Significance (* $p < 0.05$; ** $p < 0.01$; *** $p < 0.001$) difference in mean value at reassessment (21 wpc) relative to the first exposure (18 wpc) is provided for all three treatment groups as determined by uncorrected Fisher's LSD comparison tests. Significance ($p < 0.05$) following family-wise multiplicity adjustment (Sidak test; $\alpha = 0.05$) is indicated in bold. **(A)** $\dot{M}O2max$ = maximum metabolic rate; **(B)** SMRq0.2 = standard metabolic rate (0.2-quartile method); **(C)** AAS = absolute aerobic scope; **(D)** FAS = factorial aerobic scope; **(E)** EPOC = excess post-exercise oxygen consumption; **(F)** EPOCdur = EPOC duration; **(G)** RMR = routine metabolic rate; **(H)** T0.5$\dot{M}$O2max = time spent above 50% of $\dot{M}$O2max; **(I)** T0.5$\dot{M}$O2max = time spent above 80% $\dot{M}$O2max; **(J)** O2crit = critical oxygen level, **(K)** ILOS = incipient lethal oxygen saturation; **(L)** SOD = scope of oxygen deficit; **(M)** FSOD = factorial SOD; **(N)** AOD = accumulated oxygen deficit.

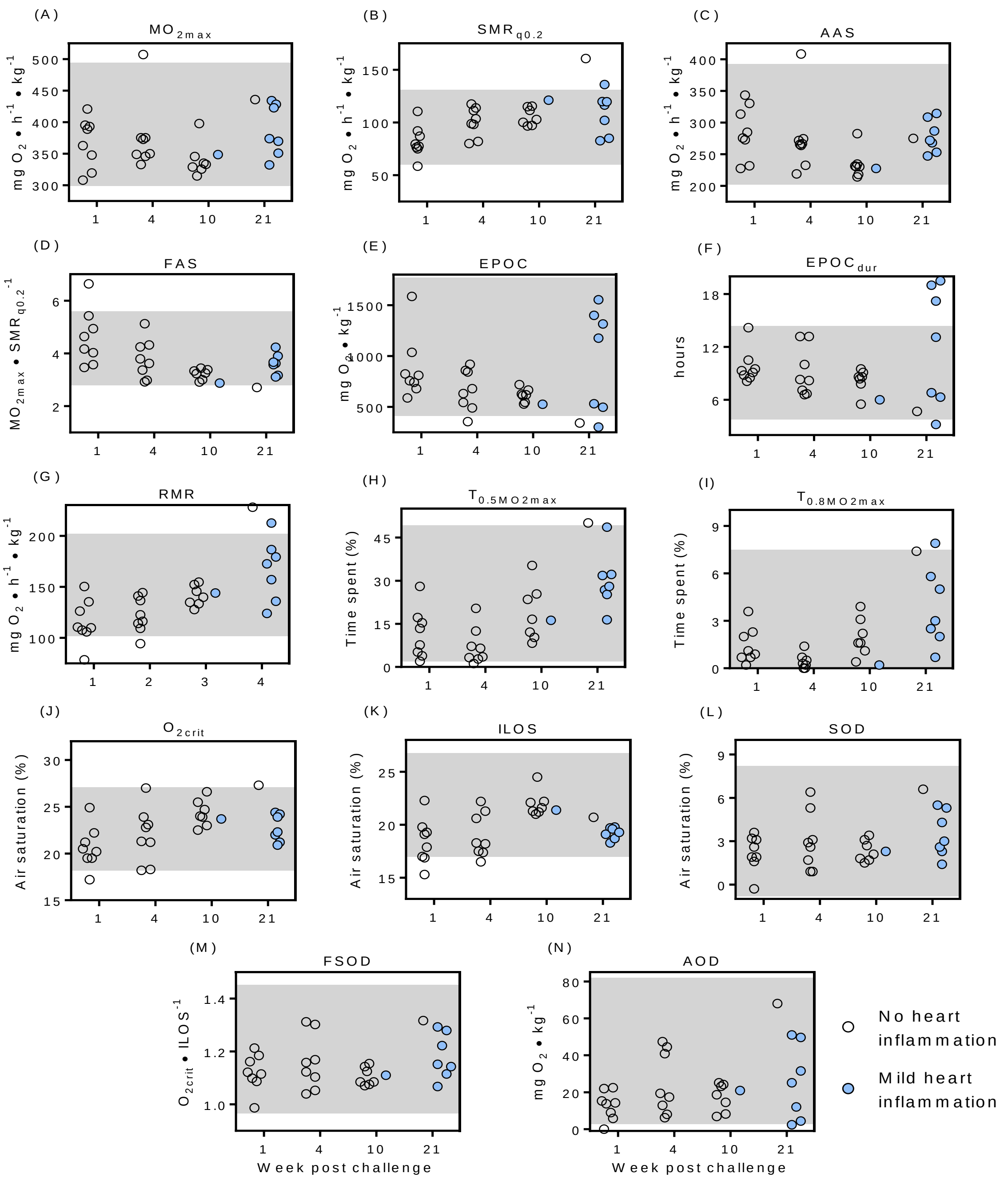

Supplementary Figure 10. The effect of heart inflammation on BC Atlantic salmon respiratory performance. Dot plots of 14 respiratory indices measured for fish exposed to an IRAP and then evaluated for heart inflammation by histoathology. No significant difference in mean value between BC fish with mild heart inflammation (blue; n=8) and fish without heart inflammation (white; n=24) were observed independent of time. Shaded area encompasses the index variability observed in SC fish over sample period. **(A)** ṀO2max = maximum metabolic rate; **(B)** SMRq0.2 = standard metabolic rate (0.2-quartile method); **(C)** AAS = absolute aerobic scope; **(D)** FAS = factorial aerobic scope; **(E)** EPOC = excess post-exercise oxygen consumption; **(F)** EPOCdur = EPOC duration; **(G)** RMR = routine metabolic rate; **(H)** T0.5ṀO2max = time spent above 50% of ṀO2max; **(I)** T0.5ṀO2max = time spent above 80% ṀO2max; **(J)** O2crit = critical oxygen level, **(K)** ILOS = incipient lethal oxygen saturation; **(L)** SOD = scope of oxygen deficit; **(M)** FSOD = factorial SOD; **(N)** AOD = accumulated oxygen deficit.

1 Methodologies for data measurement and indices calculation

1.1 qPCR threshold-cycle (Ct)

qPCR analyses were performed using a StepOne-Plus real-time detection system (Applied Biosystems) with primers and TaqMan probe targeting the L1 fragment of the PRV genome as previously described (Polinski et al., 2019). Each reaction contained 400 nM primers and 300 nM TaqMan probe, 1X TaqMan Universal Master Mix and 1 µL cDNA template within a 15 µL reaction. Cycling conditions included an initial incubation of 95 °C for 10 min followed by 40 cycles of 95 °C for 10 s and 60 °C for 20 s. Fluorescence was recorded at the end of each 60°C step. Samples were assayed in duplicate and were considered positive if both technical replicates reported a Ct value < 40 cycles. The fluorescence threshold (Ct) was manually set at a 0.01 change in normalized reporter value (ΔRn) relative to baseline signal.

1.2 PRV single-stranded (mRNA) absolute quantity

Absolute PRV quantification was determined by serial dilution of a 482 bp double-stranded DNA gBLOCK fragment (Integrated DNA Technologies) that included PRV L1 sequence targeted by the qPCR primer and probe (Garver et al., 2016). A seven-step 10-fold dilution series of the gBLOCK fragment spanning a dynamic range of 10-10^7 target copies per reaction was incorporated in duplicate into each qPCR run. For specific exclusion of double-stranded genomic PRV RNA detection, sample RNA was not heated (denatured) above 90°C prior to reverse-transcription (Polinski et al., 2019)

1.3 PRV double-stranded (gRNA) absolute quantity

Absolute PRV quantity of total PRV RNA (both mRNA and gRNA) was determined as for mRNA, except that RNA was denatured at 95°C for 5 min prior to reverse-transcription (Polinski et al., 2019). The quantity of mRNA was subtracted from this total quantity of RNA quantity to derive the dsRNA component.

1.4 Gene expression based on qPCR Ct values

A portion of RNA from each sample was treated with DNase I (Life Technologies), cleaned and concentrated using RNeasy MinElute Cleanup (Qiagen), and 1 µg reverse transcribed using a High Capacity cDNA Reverse Transcription Kit (Life Technologies) as previously described (Polinski et al., 2016). Real-time qPCR analyses were conducted on StepOne-Plus real-time detection systems using SYBR green chemistry. Each PCR reaction consisted of 2X SYBR mastermix (Life Technologies), forward and reverse primers [500 nM each; (Mikalsen et al., 2012; Svingerud et al., 2012)], and 1 µL cDNA template (diluted 1:5 in water) to a final volume of 15 µL. Samples were assayed in duplicate with a five-step, fourfold dilution series of pooled cDNA included in each run to calculate amplification efficiency, linearity, and provide inter-run calibration where necessary. Cycling conditions consisted of an initial activation of DNA polymerase at 95 °C for 10 min, followed by 40 cycles of 5 s at 95 °C, 20 s at 60 °C, and 10 s at 72 °C. Melt curve analyses were subsequently performed to ensure amplification specificity. A Ct value was assigned for each gene in each sample by manually adjusting the threshold to 0.05 change in ΔRn relative to baseline signal. A normalized relative quantity was calculated in reference to β-actin using the $2^{-\Delta\Delta Ct}$ method (Livak and Schmittgen, 2001).

1.5 Haemoglobin concentration (Hb)

Hb was determined by the cyan-methaemoglobin method using Drabkin's Reagent (Sigma-Aldrich, St. Louis, MO, USA) and a haem-based extinction coefficient of 11.01 mmol-1 cm-1 at a wavelength of 540 nm (Volkel and Berenbrink, 2000).

1.6 Partial pressure at 50% oxygen saturation (P_{50})

An oxygen equilibrium curve (OEC) was constructed for each blood sample by fitting a three-parameter logistic model to fractional Hb-O_2 saturation data. Oxygen equilibria were determined using a custom microplate-based, parallel assay, multi-cuvette tonometry cell as previously described (Lilly et al., 2013). Cuvettes were formed by placing blood samples (~ 3µL) between two sheets of low density polyethylene (Glad® ClingWrap) that were secured on an aluminum ring with two plastic O-rings, which were then placed in a gas-tight tonometry cell modified to fit into a SpectraMax 190 microplate reader (Molecular Devices, Sunnyvale, USA). Assay temperature was maintained at 11.5 ± 0.5°C and optical density (OD) was measured every 20 s at 436 nm and 390 nm, a maximum for deoxygenated Hb and an isosbestic point,

respectively. Blood was equilibrated with pure N_2 for a minimum of 30 min until OD at 436 nm was stable, which was assumed to indicate full Hb deoxygenation. Oxygen equilibria experiments were conducted at a constant CO_2 fraction of 0.25% (1.90 mmHg) with 9 stepwise increments of O_2 fraction (1.0, 2.0., 3.0, 4.0, 5.0, 7.0, 10.0, 15.0, and 21.0 % O_2, corresponding to 7.6, 15.2, 22.8, 30.4, 38.0, 53.2, 76.0, 114.0, and 159.6 mmHg) balanced with N_2 using a Wösthoff DIGAMIX® gas mixing pump (H. Wösthoff Messtechnik, Bochum, Germany). Full Hb-O_2 saturation was assumed after a tenth increment to 228 mmHg (30% O_2) in the absence of CO_2 and P_{50} was determined from the best-fit logistic model curve.

1.7 Plasma pH

Whole blood pH (approximately 200 µL) was equilibrated with 21 % O_2, 0.25 % CO_2, and balanced with N_2 in rotating glass tonometers in a 11.5 °C temperature controlled water bath. After a minimum of 1 h, blood was drawn into a syringe pre-flushed with the gas mixture, and pH was measured by drawing the blood through a Microelectrodes 16-705 flow-thru pH electrode in combination with a 16-702 flow-thru reference electrode (Microelectrodes Inc., Bedford, NH, USA) at 11.5 °C.

1.8 Hematocrit

Blood collected by caudle puncture in a 1 mL syringe and 22 ga needle was immediately used to fill a sodium-heparin treated Fisherbrand™ micro-hematocrit tube where upon one end was plugged with clay. Tubes were spun at 15,000 × g for 10 min at room temperature and packed blood cell volume was recorded as a percentage of total sample volume.

1.9 Maximum metabolic rate (MO_{2max})

$\dot{M}O_2$ measurements were calculated during each IRAP cycle from the slope of the decrease in dissolved over time provided linearity (R^2) was greater than 0.9. Typically, ~830 $\dot{M}O_2$ measurements were made per fish during the 4.5 days of IRAP. The highest observed $\dot{M}O_2$ value was assigned $\dot{M}O_{2max}$. This occurred within the first 1 to 5 $\dot{M}O_2$ measurements following exhaustion in 48 of 120 tests (40%). In the remaining 72 of 120 tests (60%), $\dot{M}O_{2max}$ appeared spontaneously during the 4.5-day IRAP monitoring period.

1.10 Standard metabolic rate ($SMR_{q0.2}$ and SMR_{L10})

We calculated SMR using two accepted methodologies: by the 0.2 quartile method [$SMR_{q0.2}$; (Chabot et al., 2016)] which reports SMR as the mean MO_2 value from the lowest 20% of all recorded MO2 values reordered during the stable monitoring period following EPOC, and by the low-10 method [SMR_{L10}; (Zhang et al., 2016)], which reports SMR as the mean of the lowest 10 $\dot{M}O_2$ values after removing the lowest 2% of the dataset. In this study, we exclusively use and report $SMR_{q0.2}$; however, we provide SMR_{L10} data as comparative reference [Zhang et al., 2019].

1.11 Absolute aerobic scope (AAS)

Calculated as $SMR_{q0.2}$ – MO_{2max} (Claireaux et al., 2005).

1.12 Factorial aerobic scope (FAS)

Calculated as MO_{2max} / $SMR_{q0.2}$ (Clark et al., 2005).

1.13 Excess post-exercise oxygen consumption (EPOC)

The area under the curve of the observed $\dot{M}O_2$ values from the end of the IRAP exhaustive chase until the observed $\dot{M}$O2 reached a MO_2 quantity of $SMR_{q0.2}$ plus 10% (Zhang et al., 2018).

1.14 Excess post-exercise oxygen consumption duration ($EPOC_{dur}$)

The time (in hours) for $\dot{M}O_2$ values to reached a quantity of $SMR_{q0.2}$ plus 10% beginning at the end of the IRAP exhaustive chase (Zhang et al., 2018).

1.15 Routine metabolic rate (RMR)

The average $\dot{M}O_2$ measure within the 4-day normoxic period following EPOC.

1.16 Time spent above 50% of MO_{2max} ($T_{0.5MO2max}$)

The percentage of $\dot{M}O_2$ measures that were at a quantity above 0.5 $\dot{M}O_{2max}$ following EPOC.

1.17 Time spent above 80% of MO_{2max} ($T_{0.8MO2max}$)

The percentage of $\dot{M}O2$ measures that were at a quantity above 0.8 $\dot{M}O_{2max}$ following EPOC.

1.18 Critical oxygen level (O_{2crit})

During the final IRAP stage of acute hypoxia, a linear regression was fitted to the period of declining $\dot{M}O_2$ against dissolved oxygen concentration for each fish. The intersection of this linear regression with $SMR_{q0.2}$ was determined as O_{2crit} (Ultsch et al., 1980; Claireaux and Chabot, 2016).

1.19 Incipient lethal oxygen saturation (ILOS)

The dissolved oxygen saturation at which loss of dorso-vetro equilibrium was observed (Claireaux et al., 2013).

1.20 Scope of oxygen deficit (SOD)

Calculated as O_{2crit} – ILOS (Zhang et al., 2017).

1.21 Factorial scope of oxygen deficit (FSOD)

Calculated as O_{2crit} / ILOS (Zhang et al., 2017).

1.22 Accumulated oxygen deficit (AOD)

Calculated as the area bounded by the $SMR_{q0.2}$ and observed $\dot{M}O_2$ lines over the time interval of O_{2crit} and ILOS (Zhang et al., 2017).

2 References

Chabot, D., Steffensen, J.F., and Farrell, A. (2016). The determination of standard metabolic rate in fishes. *Journal of Fish Biology* 88(1)**,** 81-121.

Claireaux, G., and Chabot, D. (2016). Responses by fishes to environmental hypoxia: integration through Fry's concept of aerobic metabolic scope. *Journal of Fish Biology* 88(1)**,** 232-251.

Claireaux, G., McKenzie, D.J., Genge, A.G., Chatelier, A., Aubin, J., and Farrell, A.P. (2005). Linking swimming performance, cardiac pumping ability and cardiac anatomy in rainbow trout. *Journal of Experimental Biology* 208(10)**,** 1775-1784.

Claireaux, G., Théron, M., Prineau, M., Dussauze, M., Merlin, F.-X., and Le Floch, S. (2013). Effects of oil exposure and dispersant use upon environmental adaptation performance and fitness in the European sea bass, Dicentrarchus labrax. *Aquatic toxicology* 130**,** 160-170.

Clark, T.D., Ryan, T., Ingram, B., Woakes, A., Butler, P., and Frappell, P.B. (2005). Factorial aerobic scope is independent of temperature and primarily modulated by heart rate in exercising Murray cod (Maccullochella peelii peelii). *Physiological and Biochemical Zoology* 78(3)**,** 347-355.

Garver, K.A., Johnson, S.C., Polinski, M.P., Bradshaw, J.C., Marty, G.D., Snyman, H.N., et al. (2016). Piscine orthoreovirus from western North America is transmissible to Atlantic salmon and Sockeye salmon but fails to cause Heart and Skeletal Muscle Inflammation. *PLoS ONE* 11(1)**,** e0146229.

Lilly, L.E., Blinebry, S.K., Viscardi, C.M., Perez, L., Bonaventura, J., and McMahon, T.J. (2013). Parallel assay of oxygen equilibria of hemoglobin. *Analytical biochemistry* 441(1)**,** 63-68.

Livak, K.J., and Schmittgen, T.D. (2001). Analysis of Relative Gene Expression Data Using Real-Time Quantitative PCR and the 2−ΔΔCT Method. *Methods* 25(4)**,** 402-408.

Mikalsen, A.B., Haugland, O., Rode, M., Solbakk, I.T., and Evensen, O. (2012). Atlantic Salmon Reovirus Infection Causes a CD8 T Cell Myocarditis in Atlantic Salmon (*Salmo salar* L.). *PLoS ONE* 7(6)**,** e37269.

Polinski, M.P., Bradshaw, J.C., Inkpen, S.M., Richard, J., Fritsvold, C., Poppe, T.T., et al. (2016). De novo assembly of Sockeye salmon kidney transcriptomes reveal a limited early response to piscine reovirus with or without infectious hematopoietic necrosis virus superinfection. *BMC genomics* 17(1)**,** 848.

Polinski, M.P., Marty, G.D., Snyman, H.N., and Garver, K.A. (2019). Piscine orthoreovirus demonstrates high infectivity but low virulence in Atlantic salmon of Pacific Canada. *Scientific reports***,** 40025. doi: 10.1038/s41598-019-40025-7

Svingerud, T., Solstad, T., Sun, B., Nyrud, M.L.J., Kileng, Ø., Greiner-Tollersrud, L., et al. (2012). Atlantic Salmon Type I IFN subtypes show differences in antiviral activity and cell-dependent expression: evidence for high IFNb/IFNc–producing cells in fish lymphoid tissues. *The Journal of Immunology***,** 1201188.

Ultsch, G.R., Ott, M.E., and Heisler, N. (1980). Standard metabolic rate, critical oxygen tension, and aerobic scope for spontaneous activity of trout (Salmo gairdneri) and carp (Cyprinus carpio) in acidified water. *Comparative Biochemistry and Physiology, A* 67(3)**,** 329-335.

Volkel, S., and Berenbrink, M. (2000). Sulphaemoglobin formation in fish: a comparison between the haemoglobin of the sulphide-sensitive rainbow trout (Oncorhynchus mykiss) and of the sulphide-tolerant common carp (Cyprinus carpio). *Journal of Experimental Biology* 203(6)**,** 1047-1058.

Zhang, Y., Claireaux, G., Takle, H., Jørgensen, S., and Farrell, A. (2018). A three-phase excess post-exercise oxygen consumption in Atlantic salmon Salmo salar and its response to exercise training. *Journal of fish biology* 92(5)**,** 1385-1403.

Zhang, Y., Mauduit, F., Farrell, A.P., Chabot, D., Ollivier, H., Rio-Cabello, A., et al. (2017). Exposure of European sea bass (Dicentrarchus labrax) to chemically dispersed oil has a chronic residual effect on hypoxia tolerance but not aerobic scope. *Aquatic Toxicology* 191**,** 95-104.

Zhang, Y., Timmerhaus, G., Anttila, K., Mauduit, F., Jørgensen, S.M., Kristensen, T., et al. (2016). Domestication compromises athleticism and respiratory plasticity in response to aerobic exercise training in Atlantic salmon (Salmo salar). *Aquaculture* 463**,** 79-88.
